# AI-driven robotic chemist for autonomous synthesis of organic molecules

**DOI:** 10.1126/sciadv.adj0461

**Published:** 2023-11-01

**Authors:** Taesin Ha, Dongseon Lee, Youngchun Kwon, Min Sik Park, Sangyoon Lee, Jaejun Jang, Byungkwon Choi, Hyunjeong Jeon, Jeonghun Kim, Hyundo Choi, Hyung-Tae Seo, Wonje Choi, Wooram Hong, Young Jin Park, Junwon Jang, Joonkee Cho, Bosung Kim, Hyukju Kwon, Gahee Kim, Won Seok Oh, Jin Woo Kim, Joonhyuk Choi, Minsik Min, Aram Jeon, Yongsik Jung, Eunji Kim, Hyosug Lee, Youn-Suk Choi

**Affiliations:** ^1^ Samsung Advanced Institute of Technology, Samsung Electronics Co. Ltd., 130 Samsung-ro, Yeongtong-gu, Suwon-si, Gyeonggi-do 16678, Republic of Korea.; ^2^ Department of Mechanical Engineering, Kyonggi University, 154-42, Gwanggyosan-ro, Yeongtong-gu, Suwon-si, Gyeonggi-do, 16227, Republic of Korea.; ^3^ School of Mechanical Engineering, Gyeongsang National University, 501, Jinju-daero, Jinju-si, Gyeongsangnam-do, Republic of Korea.; ^4^ School of Business Administration, Chung-Ang University, 135, Seodal-ro, Dongjak-gu, Seoul 06973, Republic of Korea.; ^5^ College of Information and Communication Engineering, Sungkyunkwan University (SKKU), 2066, Seobu-ro, Jangan-gu, Suwon-si, Gyeonggi-do 16419, Republic of Korea.

## Abstract

The automation of organic compound synthesis is pivotal for expediting the development of such compounds. In addition, enhancing development efficiency can be achieved by incorporating autonomous functions alongside automation. To achieve this, we developed an autonomous synthesis robot that harnesses the power of artificial intelligence (AI) and robotic technology to establish optimal synthetic recipes. Given a target molecule, our AI initially plans synthetic pathways and defines reaction conditions. It then iteratively refines these plans using feedback from the experimental robot, gradually optimizing the recipe. The system performance was validated by successfully determining synthetic recipes for three organic compounds, yielding that conversion rates that outperform existing references. Notably, this autonomous system is designed around batch reactors, making it accessible and valuable to chemists in standard laboratory settings, thereby streamlining research endeavors.

## Teaser

An AI-based robot capable of goal-specific dynamic optimization of molecular synthesis recipes is demonstrated.

## INTRODUCTION

The discovery of functional organic materials has led to the emergence of various organic counterparts of electronic devices, such as light-emitting diodes, complementary metal-oxide semiconductor image sensors, and solar cells, with the ongoing challenge of improving their properties. Traditionally, this endeavor has relied on a time-consuming and inefficient trial-and-error approach involving repetitive cycles of molecular design, synthesis, and characterization processes. Recognizing the need for innovation in this methodology, notable efforts spanning decades have aimed to revamp the approach. However, it is time-consuming and inefficient; thus, efforts have been dedicated for decades to innovate this methodology. In the realm of molecular design, the advent of high-throughput computational screening, supported by large-scale first-principles simulations and machine learning, marked a transformative shift aimed at reducing reliance on human knowledge and intuition and minimizing the likelihood of unexpected discoveries (*1*–*4*). The drive to streamline laborious experiments gained momentum with the onset of the electronics era, ushering in precise and accessible control over unit operations, such as dispensing, reactions, sample preparation (sample-prep.), work-up, purification, and analysis (*5*–*7*). Ultimately, the aspiration for comprehensive laboratory automation initially found its roots in the life sciences field during the 1980s (*8*), and substantial progress has been made over the past few decades (*9*–*13*). This trend toward automation has also manifested itself in the field of chemistry.

The advancement of artificial intelligence (AI) technologies in the 2010s, coupled with the availability of large-scale datasets, gave rise to the concept of robot chemists, where AI serves as the cognitive brain and the robot acts as the physical body, enabling autonomous chemical research. Challenges have persisted in the development of organic molecules using universal synthetic platforms, particularly in fields such as pharmaceuticals and biology (*14*, *15*). Notably, there has been a recent surge in the adoption of flow-based systems (*16*–*21*) due to their cost effectiveness and the ease with which processes can be controlled through configurable fluidic circuits with valves and pumps (*8*, *22*). These systems offer enhanced heat and mass transfer, allow for harsh reaction conditions in terms of temperature and pressure, and facilitate online analytical monitoring. However, flow chemistry faces limitations in handling poorly soluble reagents, lacks dedicated databases for automated synthesis planning, and typically lacks translatability between flow and batch chemistries (*8*). Innovative hybrid systems have been proposed (*23*–*26*) combining round-bottomed flasks for batch reactions and flow systems for chemical transport. Nevertheless, they encounter challenges in handling solid reagents containing metallic elements used in electronics applications. Hence, batch-type synthesis remains practical for chemists, despite its larger footprint and higher cost, due to its status as a standard protocol in mass production and development. While there have been some instances of bio-applications (*10*, *27*), constructing a batch-type automated system by integrating various hardware and software components is complex, resulting in only a limited number of studies with restricted capabilities (*12*, *28*–*31*).

**Fig. 1.**
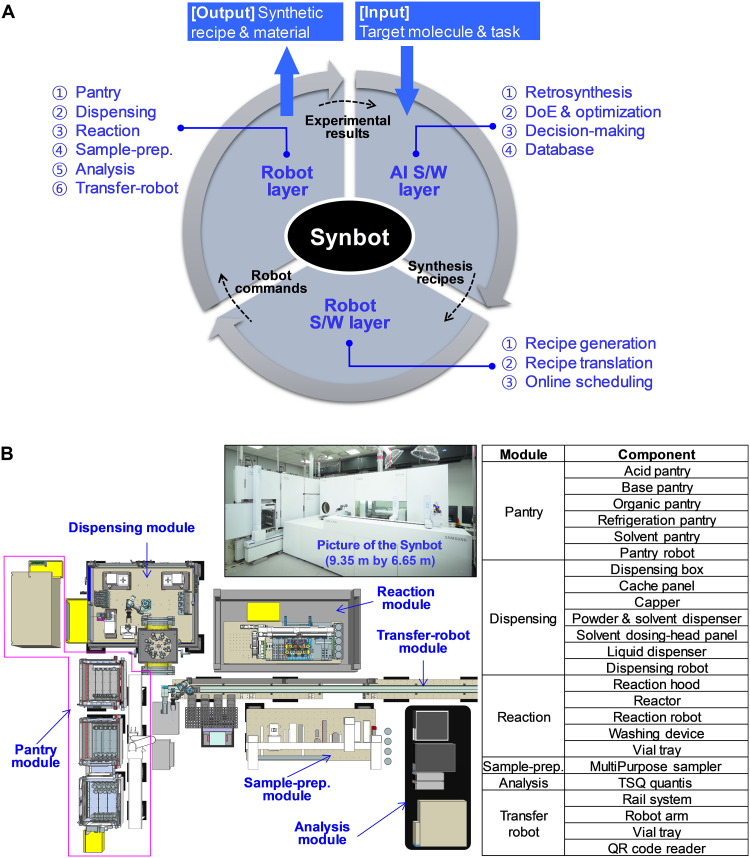
AI-driven robotic chemist (Synbot). (**A**) Structure and working concept of the Synbot comprising AI S/W, robot S/W, and robot layers. (**B**) Layout and configuration of the robot layer comprising six modules: pantry, dispensing, reaction, sample-prep., analysis, and transfer-robot.

In pursuit of a versatile and intelligent platform for molecule synthesis, this study introduces an AI-driven robotic chemist, capable of autonomously performing tasks spanning from synthetic planning to experiments conducted in batch reactors, capitalizing on the collaborative potential of AI and robots. This platform is aptly named the “Synbot” (synthesis robot). The Synbot comprises three distinct layers: an AI software (S/W) layer, a robot S/W layer, and a robot layer (Fig. 1A). Its primary objective is to synthesize target substances while actively seeking optimal conditions. The AI S/W layer spearheads the synthesis planning process, equipped with the retrosynthesis module, the design of experiments (DoE), and optimization module, and steers the direction of experiments using the decision-making module. This layer adopts a blackboard architecture, enabling individual modules to access a shared database, facilitating communication and collaborative problem solving. Once the synthesis recipe is relayed from the AI S/W layer, the robot S/W layer takes charge, translating it into actionable commands for the robots through the recipe generation module and the translation module. Subsequently, the robot layer operates under the supervision of the online scheduling module (Fig. 1B). The robot layer modularizes the various functions of the synthetic laboratory and systematically executes the planned recipes, continuously updating the database until the predefined goals are met. The Synbot encompasses essential modules, including pantry, dispensing, reaction, sample preparation, analysis, and transfer-robot modules, with an overall footprint measuring 9.35 m by 6.65 m. This comprehensive integration of AI and robotics represents a significant step toward achieving a versatile and autonomous smart synthesis platform for molecules.

### Autonomous workflow of the Synbot

**Fig. 2.**
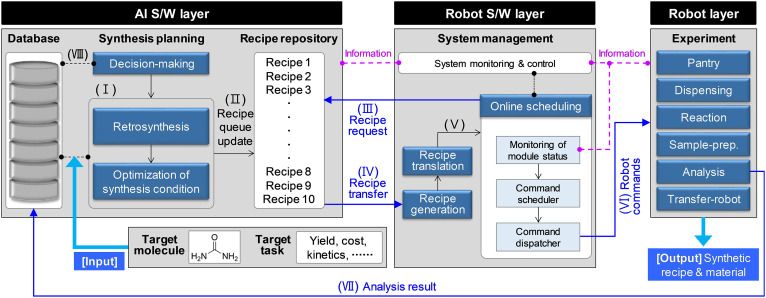
Workflow of autonomous synthesis for a target molecule and task.

The procedure for the autonomous synthesis by the Synbot is illustrated in Fig. 2. According to the target molecule and task given by a user, the AI S/W layer commences synthesis planning (i) and completes the recipe repository with the initial reaction paths and conditions (ii). When the robot S/W layer determines that one of the reactors is available, it requests a new synthesis recipe to the AI S/W layer and receives the highest-ranked recipe in the recipe repository (iii and iv). After translating the recipe into detailed robot commands (v), the online scheduler dispatches them to the robot layer (vi) when the relevant robots are prepared for execution. When analyses during the reaction are completed in the robot layer, the results are delivered to the database of the AI S/W layer (vii). The decision-making module determines whether to continue with the current recipe, to try another recipe, or to switch to a new synthetic path. The current recipe continues if the decision-making module determines that the reaction requires more time. If the decision-making module evaluates that the current recipe is not suitable to meet the target, it issues a “Withdraw” signal to the robot S/W layer to halt the current reaction condition and commence a new one. Furthermore, a “Sweep” signal is addressed to the robot S/W to stop all recipes belonging to the current synthetic path when the decision-making module concludes that another synthetic route should be attempted. The DoE and optimization module update its AI model, if the current recipe ends normally, and revise the recipe repository. Thereafter, the entire procedure repeats until the synthetic objective is satisfied.

The target task of the Synbot is currently focused on the maximization of the reaction yield. However, it can be extended to other objectives, such as the minimization of synthetic cost or the optimization of reaction kinetics, if necessary. Furthermore, in addition to the above autonomous mode, it can be operated in a semi-autonomous mode that determines optimal conditions using only the Bayesian optimization (BO) algorithm for areas not covered by deep learning models and in an automation mode that only passively performs user-specified experiments.

### AI S/W layer

Competent synthetic planning can save time and cost when obtaining a product by determining suitable combinations of starting materials and reaction conditions. The design of synthetic pathways and determination of suitable reaction conditions for a target molecule are traditionally conducted on the basis of chemists’ knowledge and experience. However, advancements in high-performance computing and AI have facilitated computer-assisted synthetic planning. While precision and validity may not yet meet the expectations of researchers, particularly for newly discovered materials, computer-assisted planning reveals implicit information from a vast body of previous studies and rapidly suggests feasible conditions. Consequently, a computer-assisted approach proves indispensable for an autonomous synthetic platform For the Synbot, a collaborative retrosynthesis approach is formulated by combining the template-based model (*32*) and the template-free tied-two-way transformer (*33*) to increase the viability of the proposed synthetic routes, which increases the top 1 prediction accuracy by 4.5 to 7.0%. When the synthesis path is determined by the retrosynthesis module, suitable reaction conditions are suggested by the DoE and optimization module (*34*) in the predefined search space (see Supplementary Text). If the target synthesis is within the material database in the AI S/W layer, then message-passing neural networks (MPNNs) (*35*) can steer the optimization process readily based on previous knowledge. However, if the task is rare, then fresh or peculiar access is crucial for reaching a solution. To address both these cases, a hybrid-type dynamic optimization (HDO) model, which associates MPNNs in conjunction with BO (*34*), is implemented to coordinate exploitation and exploration harmoniously. Various deep neural network models of the Synbot were built on the basis of the commercial Reaxys DB (Elsevier, Aalborg, Denmark). Details of the AI S/W layer are provided in Supplementary Text.

### Robot S/W layer

The synthetic recipes predicted by the AI S/W layers are abstract and cannot drive the robot; thus, they are transformed into more definite robot commands in two steps by the recipe generation and translation modules in the robot S/W layer. First, the recipe generation module produces quantified action sequences that reflect the molecular weight, purity, and concentration of the chemicals. Subsequently, the recipe translation module converts the action sequences into robot commands using concrete parameters for hardware control. The action sequences are independent of H/W configurations and are human-readable; however, the robot commands are specific to the Synbot. The online scheduling module monitors the robots’ work status in real-time and executes the commands in order (see Supplementary Text).

### Robot layer

The robot layer executes the commands received. The chemical containers of reactants and reagents, which are stored in five types of pantries (acid, base, organic, refrigeration, and solvent), are transferred to the dispensing module by the pantry robot, and subsequently, the chemicals are dispensed into glass reaction vials, as specified in the recipes. The vials were delivered to the reaction module and subjected to specific temperatures and stirring conditions for the chemical reaction. The reaction status is monitored via repetitive sampling of a small amount of the reaction solution (20 to 25 μm). The sampled solutions are then moved to the sample-prep. module and injected into a liquid chromatography–mass spectrometer (LC-MS; TSQ Quantis; Thermo Fisher Scientific, Waltham, MA). The sample-prep. module is responsible for preprocessing the sampled reaction solutions, such as dilution, mixing, and filtration of solid particles, and the final injection into the LC-MS. Each module, with the exception of the analysis module, has its own robot to handle operations, and the transfer-robot module relays the entire process by transporting the reaction and sample vials between the different modules.

We engineered the system to be robust against variations in the surrounding environment, ensuring stable operation and reliable experimental outcomes. The Synbot laboratory was under the control of a thermo-hygrostat, maintaining a temperature of ≤24°C and a relative humidity of ≤45%, thus ensuring a consistent reaction environment. In addition, the interior of the pantry and dispensing modules was continuously supplied with nitrogen gas to prolong the shelf life of the chemicals. The dispensing module was equipped with several devices for the accurate mixing of the reaction solutions. This module included a capper, dispenser for powders and solvents, dispenser for liquid chemicals, ionizer to remove static electricity, and other supporting devices. In automated systems focused on optimizing synthetic recipes, a significant portion of the experimental time is dedicated to the actual chemical reactions. Therefore, if the other devices remain idle during this phase, then it can lead to reduced overall system utilization. To prevent this, the Synbot’s reactor features six reaction slots, allowing simultaneous and independent control of multiple reactions. In addition, to avoid excessive pressure increase and solvent loss during the reaction process, a condensing mechanism and custom-built cap were applied to the reaction vial. Although LC-MS is primarily used to determine the conversion yield, it can also be used to determine the reaction kinetics. This versatility enables the Synbot to be applied to various tasks, including the mitigation of side reactions, elucidating reaction mechanisms, and developing previously unknown synthesis methods. To maintain a contamination-free operation, the Synbot extensively uses disposable glassware and devices. Further details can be found in Supplementary Text.

### Reproducibility of the Synbot

**Fig. 3.**
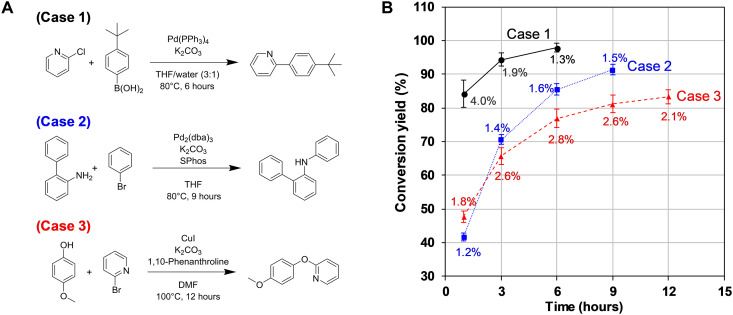
Experiments to validate reproducibility of the Synbot. (**A**) Three reaction schemes. (**B**) Conversion yield variations with time. Each number in the data point indicates the SD obtained from 12 repetitions. THF, tetrahydrofuran; DMF, *N*,*N*′-dimethylformamide.

Various factors, such as the accuracy of dispensing, consistency of the environment and chemicals, uniformity of the reaction temperature, and mixing, can influence the chemical reaction. If these factors are uncontrollable, then the reliability of the synthesis results may decrease, resulting in inaccurate outcomes. Furthermore, the generated data can negatively affect the chemical database that could otherwise have been used for machine learning. In this regard, the experimental reproducibility of the Synbot in terms of dispensing and conversion yield was examined for three typical aromatic coupling reactions (Suzuki coupling, Buchwald reaction, and Ullmann reaction; Fig. 3 and Supplementary Text).

Identical experiments were conducted 12 times to assess the reproducibility of each reaction scheme. As summarized in table S7, the chemical dispensing is carried out precisely with mean absolute errors ≤ 0.73 mg and coefficients of variance (CVs) ≤ 2.55%. In the case of the conversion yield, which reflects the consistency of all process variables including dispensing, reaction, preprocessing, and analysis, the CV values were less than 5% throughout the monitoring time. Moreover, if it is limited only to the latter part of the reaction stage, where the conversion yield converges, the CV values decrease to less than 2.5%. These results validate the performance of the Synbot and can serve as a basis for the Synbot to be used as a common synthesis platform.

### Autonomous synthesis of the Synbot

**Fig. 4.**
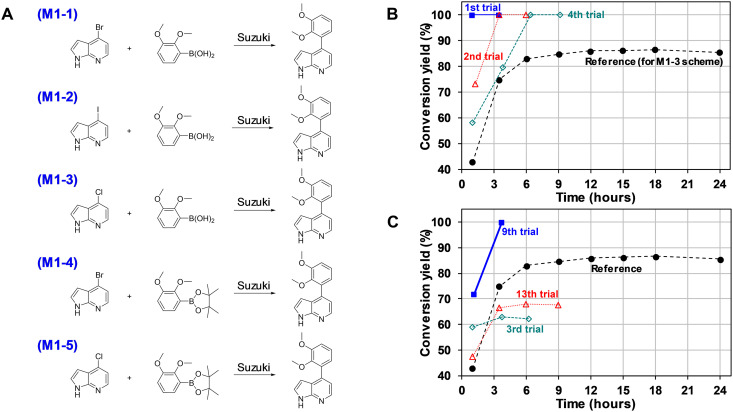
Autonomous synthesis of M1 [4-(2,3-dimethoxyphenyl)-1H-pyrrolo[2,3-b]pyridine]. (**A**) Synthetic schemes designed by AI. (**B**) Conversion yield with time for the reaction scheme M1-1. (**C**) Conversion yield with time for the reaction scheme M1-3.

**Fig. 5.**
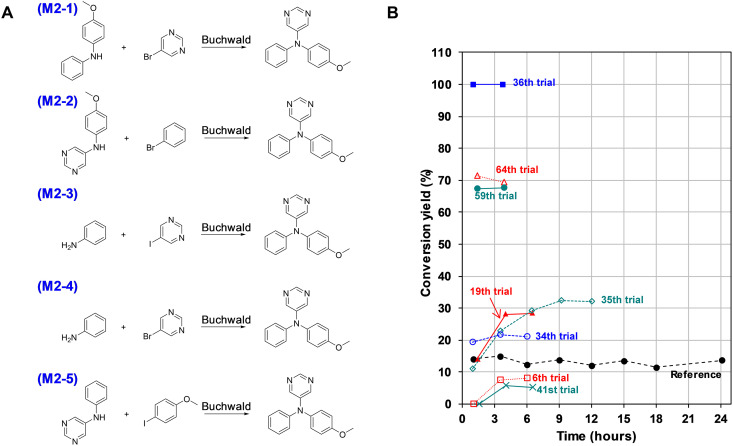
Autonomous synthesis of M2 [*N*-(4-methoxyphenyl)-*N*-phenylpyrimidin-5-amine]. (**A**) Synthetic schemes designed by AI. (**B**) Conversion yield with time for the reaction scheme M2-1.

**Fig. 6.**
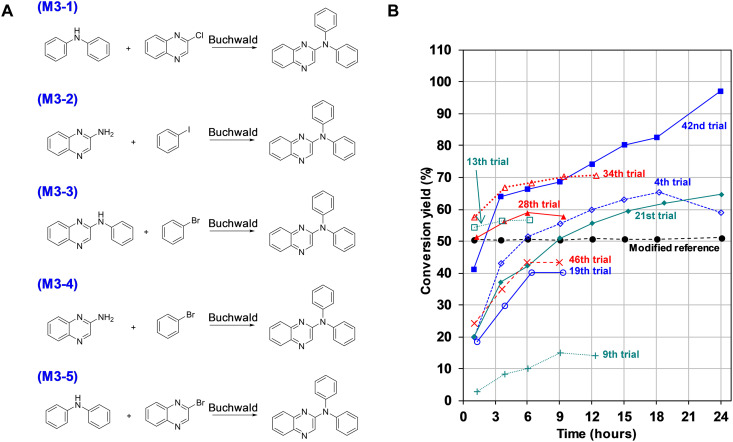
Autonomous synthesis of M3 (*N*,*N*-diphenylquinoxalin-2-amine). (**A**) Synthetic schemes designed by AI. (**B**) Conversion yield with time for the reaction scheme M3-1.

The performance of the autonomous synthesis of the Synbot was investigated using three molecules [4-(2,3-dimethoxyphenyl)-1H-pyrrolo[2,3-b]pyridine, M1; *N*-(4-methoxyphenyl)-*N*-phenylpyrimidin-5-amine, M2; and *N*,*N*-diphenylquinoxalin-2-amine, M3], which were selected from the literature (*36*–*38*) and reported to have isolation yields ranging from 30 to 50%. In advance, the information regarding the target molecules was excluded from the AI training datasets. The reaction conditions reported in the literature were reproduced on the Synbot to obtain the reference conversion yields. The results of the autonomous synthesis of the target products are summarized in Figs. 4 to 6 and tables S14 to S17.

## RESULTS

### Synthesis of M1

The reference Suzuki coupling reaction for M1 (M1-3 in Fig. 4A) (*36*) is predicted as the third priority by the retrosynthesis model, while the same synthetic routes as those found in the literature (M2-1 in Fig. 5A and M3-1 in Fig. 6A) (*37*, *38*) are proposed as the first-ranked options for M2 and M3. The reference reaction condition for M1-3 revealed a conversion yield of 86.5% on the Synbot, which is higher than the reported isolation yield of 37.7%. This discrepancy could potentially be attributed to variations in the purification step. However, it is important to acknowledge that even with the same recipe, differences in the experimental apparatus, raw materials, and environmental conditions can lead to distinct outcomes due to variations in mechanical and chemical characteristics. Therefore, a target conversion yield of 91.5%, which is 5% higher than that of the reference, was set for M1 synthesis.

Autonomous synthesis initially follows the reaction scheme M1-1, as described in Fig. 4B and table S14. Although the target yield was as high as 91.5%, a synthetic condition with a conversion yield of 100% was found in the first trial within the search space of 2722 cases. The preference of scheme M1-1 over M1-3 is readily predictable because bromine substituents are generally more reactive than chlorine substituents. However, to confirm this, an experiment for the M1-3 reaction was also conducted, as shown in Fig. 4C and table S15, and a more superior condition than the reference was obtained in the ninth trial. During this process, the Synbot learned that the tetrahydrofuran/water mixture solvent is not favorable and expanded the candidate solvents to include a toluene/ethanol/water mixture and *N*,*N*′-dimethylformamide. Furthermore, it explored different ligands and catalysts beyond Pd(PPh_3_)_4_, ultimately achieving perfect conversion using the combination of Pd_2_(dba)_3_ and BrettPhos combination. After the ninth trial, the search was continued arbitrarily to further investigate the impact of different reagents, revealing that palladium catalyst sources with dibenzilideneacetone, BrettPhos, nonstrong bases, and toluene/ethanol/water were the optimal conditions for the reaction.

To exemplify the power of the AI model, let us delve into the Suzuki coupling reaction case, denoted as M1-3. In this case, conventional catalyst and base combinations, specifically Pd(PPh_3_) _4_ and K_2_CO_3_, yielded relatively lower conversion rates within our mild temperature setup. However, under the same temperature conditions, we discovered that the less commonly used reagent combination comprising Pd_2_(dba)_3_, BrettPhos, and KOAc achieved complete reaction conversion. Notably, historical data in Reaxys DB reveal that the base and catalyst ligand we used in this case are used at only about 1% of the frequency compared to Pd(PPh_3_)_4_ or K_2_CO_3_.

### Synthesis of M2

A common problem encountered in applying AI to molecular synthesis is the scarcity of training data, a limitation driven by the vastness of the chemical space and the high cost associated with experimental data collection. In such scenarios, it becomes crucial to effectively balance both exploitation and exploration strategies. The synthetic task for M2 belongs to this category. A total of 158,609 (19.5%) of the 814,687 data used for the training of the prediction model of reaction conditions are Suzuki coupling–related data, while only 17,705 data (2.2%) belong to Buchwald amination (see Supplementary Text). Consequently, it is anticipated that discovering suitable conditions for Buchwald amination would pose a more significant challenge compared to Suzuki coupling when relying on the HDO model for exploitation.

The conversion yield of the reference M2 recipe (*37*) was only 15.0% for the Synbot (Fig. 5B and table S16). However, the yield was quantified using LS-MS (as described in eq. S1 in Supplementary Text), which can vary depending on the material’s absorbance properties. Therefore, the target conversion yield was set at 70.0%, approximately twice the reported isolation yield. For the M2-1 scheme, most recipes initially exhibited insufficient reactivity. Over time, new recipes were explored, primarily focusing on catalysts and solvents. Eventually, a combination of two types of palladium dibenzylideneacetone (dba) catalysts, Pd(dba)_2_ and Pd_2_(dba)_3_, APhos ligand, NaOtBu base, and toluene solvent was discovered, resulting in a 100.0% reaction conversion at the 36th and 37th tryouts. Autonomous synthesis continued to elucidate the reaction characteristics in greater detail, leading to more frequent proposals of high-yield reaction conditions. Through these endeavors, it became evident that bulky electron-rich dialkylbiaryl phosphine ligands are less suitable for the reaction compared to simpler monodentate or bidentate ligands such as PtBu_3_, APhos, and XantPhos.

### Synthesis of M3

The synthesis of M3 was classified as the N-arylation of Buchwald amination, as shown in Fig. 6A. However, the ligand specified in the reference literature, 2-[1,3-bis(dicyclohexylphosphanyl)-1H-inden-2-yl]-*N*,*N*-dimethylaniline, was not accessible. Consequently, XPhos was chosen as an alternative since it has been previously reported to induce rapid conversion and excellent yields, similar to the reference ligand in the same literature (*38*). The conversion yield of this modified reference condition on the Synbot was 50.9%; however, the target yield was 80.0%, considerably higher than the reported isolation yield of 45.0%.

Unlike the commonly used strong bases, such as NaOtBu, the Synbot identified high conversion conditions using the milder base of Cs_2_CO_3_ (Fig. 6B and table S17). The initial three groups suggested by the MPNN model failed to yield good results; however, the subsequent three recipes from the maximin Latin hypercube sampling (see Supplementary Text) exhibit the possibility of yielding good results. Although no clear improvement was observed until the 33rd run, the frequency of recipes with conversion yields higher than 50% gradually increased as the experiment progressed. Last, the Synbot obtained the target conversion yield in the 42nd trial using Pd(OAc)_2_ and XantPhos ligands. A closer observation indicates that a strong base, NaOH, can accelerate kinetics such as NaOtBu in the reference recipe, while Cs_2_CO_3_ results in a higher yield. Some differences were observed compared to the case of M2. First, the excellent recipes for M2 use the strong base NaOtBu, while that for M3 uses Cs_2_CO_3_. In addition, in contrast to M2 synthesis, palladium acetate performs better in M3 synthesis than palladium catalysts prepared with dba. The specificity of these reagents may be attributed to the characteristics of the reactants, quinoxaline versus pyrimidine or pure diphenylamine versus methoxy diphenylamine, with slightly different electronic structures. Although further investigation into these nuances falls beyond the scope of this work, it underscores the importance of recipe search in enhancing reaction efficiency and understanding reaction mechanisms. In this context, the utility of the Synbot can be further amplified.

To compare reference results, we conducted additional syntheses of M3 using NaOtBu, as recommended in the reference paper (*38*), in conjunction with three different ligands: tri-tert-butyl phosphine (PtBu_3_), Xphos, and Xantphos as summarized in table S18. Intriguingly, we observed that the reactions halted within just 4 hours, yielding approximately 65 to 70% conversion rates for the Xphos and Xantphos cases and a mere 5% conversion rates for the PtBu_3_ cases. While our optimal recipe exhibited a slower reaction rate, it ultimately yielded higher conversion rate.

## DISCUSSION

The customized Synbot exhibited its exceptional capabilities by consistently delivering competitive synthetic recipes with yields on par with or surpassing known references. This achievement was made possible through a closed-loop feedback mechanism between the robotic system and AI. The MPNN model effectively determined solutions for well-established Suzuki coupling reactions (M1) in a relatively straightforward, data-driven manner. Conversely, for M2 and M3, the MPNNs faced challenges in individually identifying favorable conditions but succeeded in finding solutions through collaboration with BO. The goals were achieved in all cases, with fewer than 1% of trials from the total search space, highlighting the efficiency of HDO in chemical research compared to traditional methods reliant on human expertise and knowledge. The Synbot uses not only its high-throughput experimentation capabilities but also its real-time recipe design strategy guided by AI models. This stands as a testament to the Synbot’s effectiveness in accelerating the discovery and optimization of chemical processes.

The Synbot’s ability to monitor kinetics during synthesis has the potential to enhance synthesis quality while reducing research costs. In manual experiments, the periodic inspection of reaction progress can be labor intensive, leading to reactions often proceeding for excessive durations, resulting in yield losses due to side reactions or unnecessary time wastage. The automatic analysis capabilities of the Synbot naturally address this issue. Although LC-MS provides precise quantification, its relatively lengthy and complex preprocessing is a drawback. Therefore, integrating simpler yet somewhat qualitative techniques, such as thin-layer chromatography, could enhance overall efficiency.

Depending on the total reaction time, the Synbot can conduct an average of 12 reactions within 24 hours, encompassing dispensing and analysis. Assuming a researcher can perform two experiments of this type per day, the Synbot exhibits at least sixfold increase in efficiency compared to human counterparts. This efficiency is further amplified when considering automatic synthetic planning and optimization. While the Synbot currently requires periodic human intervention to replenish chemicals, consumables like vials and filters, and dispose of waste, these challenges can be addressed by expanding pantry capacity, introducing automatic feeding robots, and implementing continuous waste-discharging mechanisms.

Efficiently assessing the properties and synthetic feasibility of materials in the early stages of development is crucial for screening potential candidates and identifying underlying issues. In this regard, the Synbot offers multiple contributions. Automated synthetic planning and decision-making guide robots empower robots to explore chemical spaces efficiently with minimal resources, enabling research even for individuals lacking extensive chemical knowledge. Accurate robot operation produces reliable experimental results, forming the basis for a high-quality DB that can be used in future studies. In addition, the Synbot provides access to numerous negative data, which are often challenging to find in typical research papers, and rich metadata for detailed causal analysis. Moreover, the batch-type reaction format aligns well with conventional synthesis practices, making it highly practical for chemists. The Synbot can accelerate the time to market for novel materials, granting researchers more time to focus on creative research activities beyond the realm of AI and robotics.

Existing chemistry DBs suffer from insufficient data and imbalance data distribution compared within the vast chemical space, leading to subpar AI performance compared to general machine learning applications like language translation or image recognition. This issue can be overcome by accelerating data accumulation through an automation platform such as the Synbot. However, ensuring compatibility of experimental results across different systems is paramount. Even batch-type reactors may exhibit variations in heating, cooling, and mixing characteristics, potentially causing discrepancies in experimental outcomes. In this respect, global standardization of experimental devices becomes imperative. Now, the Synbot is undergoing upgrades to transform to a multistep synthesis platform, including work-up and purification steps, aiming to serve as a versatile, general-purpose platform.

## MATERIALS AND METHODS

### Preparation of reagents

All reagents and starting materials were purchased from Sigma-Aldrich (Burlington, MA, USA), Tokyo Chemical Industry (Tokyo, Japan), and Daejung Chemicals (Siheung, Republic of Korea) and were meticulously prepared before storage in our laboratory’s pantries. Solid chemicals exceeding a size of 1 mm were initially subjected to grinding and sieving through a 500-μm-aperture metal sieve (TS-F0500; Glenammer, Ayrshire, UK). Subsequently, these materials were securely stored in designated chemical containers (QH010-CNMW; Mettler-Toledo, Greifensee, Switzerland), equipped with powder dispensers for convenient access. The various reaction solvents were transferred to 1-liter bottles, while nonsolvent liquid materials were carefully housed within in-house syringes. All these containers, both for chemicals and solvents, were systematically arranged in designated slots within our pantry.

### Reaction condition for autonomous synthesis

The autonomous synthesis process was executed to derive optimal reaction recipes achieving the desired target yields. This process involved navigating a four-dimensional space defined by the catalyst, ligand, base, and solvent parameters. The reaction temperature was set as a constant, contingent on the specific solvent type [for detailed temperature settings, refer to Supplementary Text (*35*, *39*–*44*)]. In all cases, the equivalent ratios of reactant 2, catalyst, and base to reactant 1 were determined from relevant literature (*36*–*38*). However, it is worth noting that due to the unavailability of equivalent ratios for the ligands in M1-1 and M1-3, ligands with twice the equivalent ratios of the catalysts were used. Concentrations for each reaction were established following established literature protocols. For comprehensive information regarding the Synbot system and the experimental procedures, please consult the Supplementary Materials.

## Supplementary Materials

### This PDF file includes:

Technical descriptions of the Synbot Procedure and results of experiments Figs. S1 to S20 Tables S1 to S18 Legend for movie S1 Legend for data S1
