# Supplementary material for: AI-driven robotic chemist for autonomous synthesis of organic molecules
Source: Sci Adv. 2023 Nov 1;9(44):eadj0461. doi: 10.1126/sciadv.adj0461 (PMC10619927; doi:10.1126/sciadv.adj0461)
Supplement: Supplementary file 1 — Technical descriptions of the Synbot Procedure and results of experiments Figs. S1 to S20 Tables S1 to S18 Legend for movie S1 Legend for data S1 [file sciadv.adj0461_sm.pdf]

Science Advances
AAAS

Supplementary Materials for

AI-driven robotic chemist for autonomous synthesis of organic molecules

Taesin Ha *et al.*

Corresponding author: Youn-Suk Choi, ysuk.choi@samsung.com

Sci. Adv. **9**, eadj0461 (2023)
DOI: 10.1126/sciadv.adj0461

The PDF file includes:

Technical descriptions of the Synbot
Procedure and results of experiments
Figs. S1 to S20
Tables S1 to S18
Legend for movie S1
Legend for data S1

Other Supplementary Material for this manuscript includes the following:

Movie S1
Data S1

Architecture of the Synbot

The Synbot was designed with a three-layer architecture, as shown in fig. S1, to provide scalability and flexibility for various types of reactions and hardware (H/W) configurations. The first is the artificial intelligence (AI) software (S/W) layer, responsible for proposing candidate reaction recipes based on AI algorithms and determining the optimal reaction conditions through experimentation iterations. The second layer is the robot S/W layer, which links the AI and robots based on recipes and manages the entire process. The third and the final layer is the robot layer, which performs all the experimental processes, starting from dispensing to analysis.

The AI S/W layer relies on machine learning, and it's crucial to synchronize all algorithms based on the same information. To achieve this, it adopts a blackboard architecture in which each functional module is directly connected to the database (DB), and all data are shared through this central hub. The AI S/W layer comprises several key modules, including a decision-making module for guiding experiment progress, a retrosynthesis module for designing synthetic paths, a design of experiment (DoE) and optimization module for refining synthetic conditions, and a DB manager.

The proposed reaction recipes generated by the AI S/W layer are then transformed into action sequences, which include specific process parameters, and translated into actual robot commands. This translation is carried out by the recipe generator and translator modules within the robot S/W layer (for more details, refer to the "Recipe generation and translation" subsection in the "Robot S/W layer" section of this document). The job scripts are subsequently dispatched to the robot layer, where they drive the hardware (H/W) modules under the supervision of the online schedule.

At present, the robot layer comprises six modules: pantry, dispensing, reaction, sample preparation (sample-prep.), analysis, and transfer robots. Detailed descriptions of these modules are provided in the following sections.

AI S/W layer

<u>Training dataset for machine learning models</u>

The Synbot adopts data-driven synthetic planning, and all the models are based on the Reaxys DB, which includes experimental chemical reaction data extracted from a vast number of studies. We reorganized the 50 million extracted data and utilized them to build the retrosynthesis and reaction condition prediction models described in the two subsections following this, respectively. Currently, the Synbot focuses on the single-step carbon-coupling reactions of C-C, C-N, C-O, and C-S; however, its capability is expandable. The reactions represented by non-element species such as R-groups were excluded. Only six reaction types were selected from the data by referring to the “reaction name” field in the DB: Suzuki coupling, Heck reaction, Sonogashira coupling, Stille reaction, Buchwald amination, and Ullmann reaction.

The reaction records, which include information on the reaction name, reactants, product, reagents, synthetic conditions, and isolated yield, were extracted to train the prediction model of suitable reaction conditions in the DoE and optimization module. In addition, the data consisting of more than two reactants or more than one product were filtered. Although it is possible to build prediction models for general types of reactions, the data imbalance of the Reaxys DB would cause low accuracy. This is the main reason for this study's limited range of synthesis methods. Finally, 211,588 and 814,687 data points were used to build the reaction conditions' retrosynthesis and prediction models, respectively.

Retrosynthesis

An ensemble retrosynthesis model is proposed by combining template-based retrosynthesis and the template-free tied-two-way transformer (TTT) models (*33*).

The template-based model follows Segler's protocol (*32*). We extracted 6,144 reaction templates from the reaction data described in the above subsection, using RDChiral (*39*), and built a deep neural network (DNN) model with the templates to predict reactants for a given product. The DNN model consists of input, output, and hidden layers. As an input for the model, the simplified molecular-input line-entry (SMILES) of the product molecule was converted into an extended connectivity fingerprint (ECFP) vector with dimensions of 8,192. The hidden layer had 256 nodes, with a dropout rate of 0.5. The output layer is a softmax-based classifier for one-hot-encoded reaction templates. The DNN model was trained using the Adam optimizer (*40*) with a learning rate of 0.001. As a loss function, cross-entropy loss was adopted to calculate the gap between the target and predicted reaction templates. The reaction dataset was split into training, validation, and test sets with a ratio of 8:1:1. After deriving the reaction pathways; their suitability was assessed using an in-scope filter, following Segler's model (*41*). We generated possible reactants for the target products to provide a negative dataset to the in-scope filter. We extracted the corresponding reaction rules using the rdchiralRunText function of the RDChiral. The case is excluded if there are more than 10 reactant combinations for one product because it can induce a bias. A pair of reactants and products is considered positive if it matches the reaction data from which the corresponding reaction rule is extracted, while all other cases are considered negative. Through this, 186,656 and 58,528 positive and negative data points were constructed, respectively.

The structure and hyper-parameters of the TTT model are the same as in a previous study (*33*), other than updating the OpenNMT-Py package version from 1.1.1 to 2.2.0 (*42*). The TTT model was trained with the 211,588 dataset described in the above subsection on four NVIDIA Tesla V100 GPUs.

In terms of numerical performance, the TTT model was superior to the template-based method, as summarized in table S1. However, it sometimes proposes implausible pathways that disobey basic physical theory, such as atomic conservation, because it is a sequence-based model. Therefore, the ensemble approach was devised by balancing the two models. After predicting each model's top 20 synthetic routes, a rescoring process was conducted to rank the routes. The softmax score of the NN in the template-based retrosynthesis and the likelihood score of the TTT model were interpreted as the probability of the prediction results. Thus, an evaluation score was defined using these two scores to assess the synthetic paths. If a synthetic path is deduced from both models, the evaluation score is calculated as the average value of the softmax and likelihood scores. However, if the path is generated from only one model, the evaluation score is defined as half the softmax or likelihood score. The top-n candidate routes are determined by re-organizing the synthetic paths in descending order of the evaluation score. Table S1 shows that the ensemble model performs better than each standalone model.

DoE and optimization

A hybrid-type dynamic optimization (HDO) (*34*) based on message-passing neural networks (MPNN) (*35*) and Bayesian optimization (BO) (*43*) was developed for efficient recipe optimization. The HDO model utilizes the information from the large-scale Reaxys DB and

current experiments and determines the priority of the next candidate recipes by controlling the weight of exploitation and exploration.

The MPNNs, multi-label classifiers trained with approximately 0.8 million reaction data points, propose a combination of catalyst, base, ligand, and solvent for a given reaction pathway, and the suggested series of reagents constitute the sources for exploitation during optimization. In addition, as a part of the exploration strategy, maximin Latin hypercube sampling (MLHS) (*44*) is used to obtain uniformly distributed random samples by maximizing the minimum distance between design points. In the initial stage of optimization, some of the experimental recipes were taken from the MPNNs, and the remaining recipes were selected with the MLHS to balance exploitation and exploration. Since the experimental results were produced for the synthetic route, the acquisition function of BO ranked the priority of the next recipes following the training of the objective function of the Gaussian process. At the end of each reaction, the HDO model dynamically weighs the MPNN and BO by considering the optimization history and finally selects the next experimental condition among the candidates. The loop continues until the optimal recipe is obtained.

Decision making

The decision-making module determines whether a particular reaction recipe is terminated based on the status of the reaction. The basic principle is to shorten the overall experimental time for optimal recipe exploration while obtaining sufficient information.

The reaction recipe ends typically with an optimal reaction condition when the conversion yield reaches the target value. However, the decision-maker sends a “kill” signal to the robot S/W layer to end the current reaction in the following three cases: if the total elapsed reaction time exceeds the setting value (generally 48 h), when the conversion yield begins to decrease, and if the increasing rate of the conversion yield is less than 0.5% per hour. The robot S/W layer then empties the corresponding reaction slot and requests a new recipe for the AI S/W layer. When the decision-making module determines that it is relatively challenging for the current synthetic path to reach the target yield, it calls the retrosynthesis module another synthetic path.

AI database

We built a MongoDB (version 3.6.9)-based DB to manage the data from the Synbot. All experimental information was stored to monitor the optimization process, and the results were reflected in the optimization model.

Robot S/W layer

Configuration of the control system

The H/W modules of the Synbot are controlled by seven process managers (PMs) as depicted in fig. S1, and each PM was built on separate industrial PCs (FPC-7902; Arbor, New Taipei City, Taiwan). The robot S/W layer that functioned as the master controller was defined as PM1. The remaining six PMs that supervise the H/W modules act as slave controllers. PM1 monitors and manages the slave controllers (PM2–7), which each have sub-schedulers (sub-SCDs) to monitor and drive H/W devices in the robot layer. The robot S/W framework was developed based on Samsung-CTC (the technical details are not described in this paper), Samsung’s S/W platform for systematic integration and control of various equipment.

Process management

The robot S/W layer receives synthetic recipes from the AI S/W layer. It hierarchically converts them into action sequences of tasks and functions to implement the abstracted recipes as actual robot actions, as described in the following subsections and fig. S2. Then, the drivers execute the functions, which is an S/W interface for controlling each device in the H/W modules. Most devices in the Synbot are each controlled by a dedicated driver; however, one driver may control some devices that operate together, for efficiency. A typical example is the transfer-robot module that runs both a rail and robot arm to transfer vials between modules.

Abstraction of experimental process

The experimental process was divided into four tasks, listed in table S2. Each task, defined as a meaningful unit of work in chemical synthesis, consists of a set of functions that should be conducted sequentially. This function refers to a unit operation in each module and becomes a building block for scheduling. For example, an uncapping function in the dispensing module (described in the "Dispensing module" subsection in the following section) to open the cap of a reaction vial starts by picking up the reaction vial and transferring it to the capper. After the capper opens, the dispensing robot transports the vial to the cache panel. This series of operations is performed by combining various driver commands to control hardware devices.

Recipe generation and translation

The synthetic recipes proposed by AI modules are unable to run H/W devices directly. Thus, the process of transformation into definite commands is necessary. For this, we devised a two-step process: recipe generation and translation, as illustrated in fig. S2.

The synthetic recipe contains general information about the reaction mechanism, which is irrelevant to the H/W configuration: target and starting materials, reagents (catalyst, base, ligand, solvent, etc.), concentration or ratio of chemicals, and temperature. The recipe-generation module creates an action sequence (fig. S2(b)) with a combination of tasks by referring to the chemical DB, which includes information such as the molecular weight, purity, and concentration of chemicals. At this step, the concentration of the reaction solution was set at 0.2 M, and the dispensing of the chemicals was defined to proceed in the order of solid, solvent, and liquid. The action sequence is still H/W-independent and is represented in plain text, enabling any researcher to understand and perform the experiment. The recipe translator then changes it into actual robot commands (functions) by considering the H/W configuration of the Synbot. In this stage, the execution order is rearranged, and the detailed motions of the devices are specified.

The actual robot commands are based on the SCTC language and directly matched to the operation unit of H/W devices. When troubleshooting, it is easier to identify the problem if commands are named in a very human-readable manner. For example, 'Transfer(Opt,Pd,Sd)' is a dispensing robot instruction that stands for the transfer command of the optimization vial (Opt) from the powder dispenser (Pd) to the solvent dispenser (Sd). Each command follows the SCTC language and, at the same time, adheres to the naming rule we have defined. This enables humans to identify and respond to problem situations as they emerge.

Online scheduling

The operation of the Synbot is challenging in terms of scheduling because unpredictable recipes are continuously generated by the AI algorithm. Therefore, unlike conventional offline

scheduling, in which all decisions are made before running, the Synbot requires a dynamic scheduler to consider real-time H/W occupancy. For this purpose, we propose a multi-agent-based scheduling (MAS) approach in which each H/W module is defined as an independent and autonomous agent that can conduct given tasks under the supervision of its sub-SCD. The main SCD in the robot S/W layer coordinates the sub-SCDs and submits feasible tasks at the appropriate time.

The scheduling procedure is illustrated in fig. S3. After the recipe is received by the AI client, the transformed tasks and functions are stored in the storage named the SCD-repository. The task scheduler then loads the sequence of tasks and dynamically dispatches them to the sub-SCDs when responsible agents become available. Each sub-SCD performs dispatched tasks independently. After the completion of each recipe, the experimental results were reported to the AI S/W layer to determine the next step of the procedure.

Process database

Various supplies, such as chemical reagents, solvents, vials, and filters, are consumed during the operation, and their inventory status is managed with the process DB for systematic replenishment.

The slots carrying reagent containers inside each pantry are assigned unique numbers. Information on the location, container type, and chemical ID with purity, phase, concentration, and residual amount is continuously updated. The Synbot uses two types of vials for reaction and analysis. Owing to the large number of vials consumed in the experiment, records such as vial type, ID, slot number in the trays they occupy, and state (used or unused) are continuously tracked. Filters in the sample-prep. module were managed similarly.

Graphical user interface

The Synbot has a master graphical user interface (GUI) installed in PM1 and six slave GUIs in PM2–7. The master GUI can monitor and control the system by communicating with the AI S/W layer and the slave controllers. Even though the master GUI has all the functions of the slave GUIs, we provided the slave GUI of each H/W module for the operator's convenience or emergencies. Fig. S4 shows an example of the master GUI screen; the remaining slave GUIs follow the same format.

Robot layer

Design concept

Many automation systems for material synthesis have been developed. They can be classified into three main types in terms of reaction types and sample transport: (1) a batch-type reaction and sample transport similar to experimental methods in common laboratories, (2) a flow chemistry that drives chemical reactions in small tubing and transports reagents through pumps and valves, and (3) a hybrid-type that combines the batch-type reaction and flow-type sample transport. Methods based on fluidics ((2) and (3)) have the advantage of relatively easy implementation and low cost, but they have critical issues – clogging and cross-contamination – in terms of solid handling and cleaning. In addition, most of the reaction data accumulated thus far are based on batch-type reactions; thus, the flow-type reaction has a limitation in data compatibility for applying AI models. In this respect, the Synbot is designed according to batch-

type reactions, and all experimental consumables are used as disposables to prevent cross-contamination.

The Synbot was designed to monitor the progress of the reaction by liquid chromatography-mass spectrometry (LC-MS) analysis. Although each analysis requires approximately 30 min to complete, this feature provides a significant opportunity to study the reaction mechanism and determine the appropriate timing for maximum yield based on kinetic information. The Synbot was installed in an air-conditioned room (temperature ≤ 24 °C, relative humidity ≤ 45%) to exclude environmental factors and can work in a dark environment to handle any light-sensitive chemicals.

Layout and experimental workflow

The Synbot comprises the following six modules for performing single-step organic synthesis and recipe optimization, as depicted in fig. S5: (A) the pantry module for stable chemical storage, (B) the dispensing module for the precise preparation of chemicals in reaction vials, (C) the reaction module where chemical reactions and periodic sampling for reaction monitoring are performed, (D) the sample-prep. module for pre-processing the analysis samples, (E) the analysis module to analyze the sample solutions by LC-MS, and (F) the transfer robot for handling vials between modules. To secure the stable operation of the transfer robot, each module is installed on sole plates whose relative distance error is 0.1 mm/m and whose height difference is within ± 0.1 mm.

The experimental workflow starts with a preparation step. (i) Chemical containers are manually placed in pre-defined positions of the pantry module. In addition, other supplies, such as reaction vials with magnetic bars, sample vials, particle filters, and solvents (for washing, dilution, and analysis), are also provided in the specified places. (ii) When a start command is reached, the transfer robot sends an empty reaction vial to the dispensing module, and the pantry robot moves the required chemicals from the pantry to the dispensing module. (iii) After dispensing the chemicals into the reaction vial, the pantry robot returns them to their original pantry positions, and the reaction vial is passed to the reaction module by the transfer robot. (iv) In the reaction module, the processed reaction vial is placed into one of the reactor slots for the chemical reaction. To monitor the reaction progress, a small portion of the reaction solution is periodically sampled by a multifunctional end-effector in the reaction robot and injected into the sample vial. The washing step of the sampling needle, tubing, and syringe pump in the reaction robot (discussed below) is followed to prevent cross-contamination. (v) Simultaneously, the transfer robot delivers the sample vial to the sample-prep. module to pretreat the samples (mixing, particle filtration, and dilution) for LC-MS. (vi) The processed sample is injected into the LC-MS and analyzed in the analysis module. The termination time of the reaction is determined according to the LC-MS results, and the above procedure is repeated until optimal conditions are obtained.

Pantry module

The pantry module stores the chemical containers in five types of pantries, as shown in fig. S6, and delivers them to the dispensing module through the pantry robot.

Pantry

Table S3 summarizes the specification of the pantries for acid, base, organic, refrigeration, and solvent. The powdered-solid and liquid chemicals are stored in powder dosing heads (QH010-

CNMW; Mettler-Toledo, Greifensee, Switzerland) and syringe containers developed for this study, respectively. The containers are stored in the pantries according to their chemical types. Organic solvents for reactions are contained in 1-L glass bottles and stored in the solvent pantry.

The acid, base, and organic pantries can maintain chemicals in nitrogen atmosphere (≥ 99% v/v) at room temperature, whereas the refrigeration pantry is controlled to approximately 4 ℃ in air. The solvent pantry maintained a nitrogen atmosphere inside the solvent bottles and transferred the solvents to the dispensing module through tubing under the control of the pump and selection valves. All pantries except the solvent pantry have automatic front doors for robot access and manual rear doors for human access.

Pantry robot

The pantry robot (PF3400; Precise Automation, Livermore, CA) transfers powder dosing heads and syringe containers between the pantry (acid, base, organic, and refrigeration) and the buffer zone of the dispensing module. A 1.16-m-high SCARA cooperative robot mounted on a 2-m rail covers a sufficient workspace in the horizontal and vertical directions. The gripper of the SCARA robot was designed by considering the shape of the chemical containers.

<u>Dispensing module</u>

The dispensing module is responsible for the quantitative dispensing of reactants and reagents into the reaction vials in a controlled environment. The operation proceeds with several active devices, such as the capper, dispensers, and dispensing robot, along with auxiliary devices. Fig. S7 depicts the layout and components of the module.

The general dispensing procedure consists of 15 steps, as shown in fig. S8. Before starting, an uncapped vial (V_0) was placed in the cache panel. (1) After opening the outer door of the buffer zone, the transfer robot loads a new capped vial (V_1) and chemical containers into the turntable of the buffer zone. Then, the outer door is closed. (2) The inner door of the buffer zone opens, and the turntable rotates to a pre-defined position to allow the dispensing robot to access V_1 and chemical containers. (3) The dispensing robot takes V_0 from the cache panel and loads it into the powder and solvent dispensers after the removal of static electricity by the ionizer. (4) The dispensing robot transfers V_1 onto the capper, and (5) the capper uncaps it. (6) uncapped V_1 is delivered to the cache panel and its cap remains in the capper. (7) Powder and (8) solvent dispensing into V_0 proceeded in order by changing the dosing heads. (9) The dispensing robot loads V_0 into the liquid dispenser. (10) The liquid dispenser dispenses liquid reactants or reagents by changing syringes. (11) The processed vial (V_0) is transported to the capper and (12) capped with the cap that was detached from V_1. (13) The dispensing robot loads capped V_0 onto the turntable. Subsequently, the inner door of the buffer zone is closed. (14) The outer door of the buffer zone opens, and the turntable rotates to allow the transfer robot to access capped V_0. (15) Finally, the transfer robot moves capped V_0 to the reaction module. The pantry robot returns the chemicals to the pantries. Then, the outer door is closed. The time for completing the total dispensing process is approximately 20–30 min, depending on the recipes.

Dispensing box

The dispensing box was designed to maintain an inert, dry environment. It controls the atmosphere (N_2 ≥ 99.7%, relative humidity ≤ 5%) through a programmable logic controller with gas, humidity, and pressure sensors. The buffer zone, which comprises the outer door, turntable, and inner door, is provided to input and output vials and chemicals with minimal disturbance to

the internal atmosphere. Therefore, it also has an atmospheric-control function which is operated at (2) step and (14) step in dispensing procedure. The transfer and dispensing robots access the buffer zone through the outer and inner doors, respectively. The turntable has temporary storage slots for eight reaction vials and eight chemical containers and pairs the slots to the robot access positions by rotation.

Cache panel

The cache panel is an additional temporary storage location for both the reaction vial and chemical containers. It can hold one reaction vial and seven chemical containers. Frequently used reagents are stored in the cache panel during dispensing to reduce delivery time. One reaction vial is stored in the cache panel to secure the natural discharging time of the static electricity induced by uncapping.

Capper

The capper is responsible for opening and closing the caps of the reaction vials before and after dispensing. The developed capper consists of two main parts, a 3-degree of freedom (DoF) cap rotator and a 1-DoF vial holder, as illustrated in fig. S9A. The cap rotator grips (gripping force of up to 10 N) and rotates (torque of up to 1.5 Nm) the vial cap by controlling the speed of the two electric motors. The 1-DoF ball-screw mechanism controlled the vertical position. During uncapping and capping, the vial holder prevents slipping of the vial by clasping (gripping force of up to 13.2 N).

The uncapping and capping processes are conducted with the cooperation of the capper and the dispensing robot, as shown in fig. S9B. The dispensing robot loads a reaction vial to the capper and maintains it until it activates. After the capper holds the vial, the dispensing robot releases it. The capper then adjusts the vertical position of the cap rotator and conducts uncapping or capping actions. After the operation, the dispensing robot takes the vial again, and the capper returns to the ready mode. The time for uncapping and capping is approximately two minutes.

Powder and solvent dispenser

A commercial Quantos (XPE205V + Q2 module + QL2 pump module; Mettler-Toledo) was used to dispense powders and solvents by mounting one of the powder dosing heads or liquid dosing heads (QL001; Mettler-Toledo), respectively. It controls the dispensing speed and accuracy with the feedback from a balance capable of weighing in the range from 0.01 mg to 200 g.

One of the critical issues in dispensing is the static electricity generated by mechanical friction. In the capper, uncapping induces static electricity in the reaction vial from the friction with the septum in a cap (as discussed in a subsection below), and the static electricity can deteriorate the dispensing accuracy. To avoid this issue, we discharged the static electricity by exposing the reaction vial to a bar-type antistatic kit (Ionizing kit Quantos system; Mettler-Toledo) for 30 s before loading it onto the dispenser. In addition, sufficient natural discharging time is ensured by using the previously uncapped vial (V_0) in cache panel instead of the recently inserted vial (V_1), as described in the previous subsection.

Solvent dosing-head panel

The 20 liquid dosing heads receive solvents from the solvent pantry through tubing and are hung on the solvent dosing-head panel before use. When the dispensing robot attempts to load one of these dosing heads to the dispenser, entanglement of the tubing can occur. To prevent this, a tubing cartridge was developed by combining a constant force spring (CFS0.1; MISUMI, Tokyo, Japan) and tubing guide, as depicted in fig. S10. The tube cartridge releases tubing naturally when a dosing head is mounted in the dispenser. In contrast, the tubing is rewound into the cartridge with the spring force when the dosing head returns to the dosing-head panel.

Liquid dispenser

A liquid dispenser was developed to dispense small volumes of liquid chemicals precisely. Many commercial liquid dispensers, including Quantos, are unsuitable for handling small volumes or difficult to apply to expensive reagents because of their sizable dead volume. Fig. S11 shows our dispenser and the syringe-type liquid container. The dispenser consisted of a locking part to hold the liquid container, an adjustment part to control the syringe's vertical position, a pressing part to push the syringe plunger, and a balance to measure the dispensed mass. The liquid container comprises of a commercial syringe (gas-tight syringe of 1 mL or 2.5 mL with a 30-gauge needle; Hamilton Company, Reno, NV) and a housing which is customized for handling by the dispensing robot.

Fig. S12 describes the sequence of liquid dispensing. After mounting a liquid container on the dispenser by the robot, the locking part grasps the liquid container. Following the vertical position adjustment between the liquid container and a reaction vial, the dispenser finely presses the plunger with feedback from the balance until a target amount is reached. The container is then handed over to the robot in reverse order.

In the validation tests with chloroform, triethylamine, 2-bromo pyridine, 2-ethoxy ethanol, and benzonitrile, the liquid dispenser showed coefficients of variation (CVs) of $\leq 2.1\%$ and mean absolute percentage errors of $\leq 2.4\%$ for target amounts of ≥ 37.7 mg (n=12, data not shown).

Dispensing robot

The dispensing robot handles reaction vials and chemical containers inside the dispensing module. The robot consists of a robot arm (UR3e; Universal Robots, Odense, Denmark) and gripper (Hand-E; ROBOTIQ, Quebec, Canada) with a gripping tip (fig. S20).

<u>Reaction module</u>

The reaction module conducts chemical reactions and periodic sampling of the reaction solutions. The reactor was equipped with a reaction robot for vial transfer and sampling, a washing device for cleaning the sampling devices, and vial tray. To maintain stable reaction performance, a reaction vial with a gas-tight cap was developed. The layout of the module is illustrated in fig. S13.

The reaction module begins when the dispensed reaction vial arrives in the vial tray. First, the reaction vial was placed into one of the reactor slots specified by the scheduler. During the reaction, the reaction robot repeatedly sampled a small amount of the solution and transferred to a sample vial. Following each sampling, the sampling needle in the robot was cleaned in the washing device to prevent cross-contamination. The sample vial was then moved to the sample-prep. module.

Reactor

The reactor had six reaction slots that can perform heating up to 250 °C, magnetic stirring at up to 2000 RPM, and condensation. The cylindrical hot-stirring part played the role of heating and stirring with a hot plate and stirring motor, as shown in fig. S14. The heat generated in the hot plate was transported to a heating block by conduction and finally applied to the lower part of the reaction vial. In the upper part of each reactor slot, a cooling block was provided to condense the evaporated solvents into the liquid state. The IKA chiller (CBC 5 control; IKA, Staufen, Germany) circulated the coolant (deionized water) through pipes, flowmeters, needle valves, and cooling blocks at a specified temperature (approximately 10 °C).

The reactor can independently control six chemical reactions without thermal interference through a zigzag arrangement of each reaction slot. The neighboring slots were 20 mm apart, with thermal insulation. All electronics and control algorithms for the monitoring and PID control of the reactor were packed in a separate control box. The temperature and stirring speed were precisely controlled by a deviation of ± 2% (data not shown), and the coolant flowed evenly through each cooling block to achieve a uniform condensing temperature (table S4).

Reaction vial

The reactor applies and extracts heat to the reaction vial’s lower quarter and upper quarter surfaces, respectively. The other half of the vial surface was in contact with atmospheric air during the reaction. For stable operation, a reaction vial consisting of a borosilicate glass vial and a cap was developed. The glass vial had a diameter of 13 mm and length of 70 mm and was designed to react with approximately 1 mL of solution.

The vial was exposed to various temperatures, internal gas pressures, and solvent fumes during the reaction. In addition, repeated needle penetration into the septum during sampling increases the risk of leakage. To withstand this harsh condition, the vial cap was specially designed, as shown in the cross-sectional view in fig. S15. The dual-capping structure with outer and inner caps secured the sealing of the silicone septa by forming tight contact between the septum edge and the lip of the vial inlet. In a test with tetrahydrofuran (THF) at 80 °C and caps of a six-times perforated septum, the solvent loss was less than 5% after 24 h.

Reaction robot

Sample manipulation in the reaction module was performed using an XYZ-stage robot (three-dimensional gantry; Festo, Esslingen am Nectar, Germany) and a multifunctional end-effector attached to it, as illustrated in fig. S16A. The developed end-effector grips the vial and the sampling and dispensing of the reaction solution during synthesis.

For the gripping action, the end-effector applies a normal force to the vial in four directions using a four-way gripper (fig. S16B). This can compensate for the misalignment and shape variation of vials, thus enabling stable handling. The gripping motor rotates the gripping frame, which is connected to the gripping gear using a force-adjusting spring. Subsequently, the gripping frame sequentially conveys the gripping force to the four-way gripper.

The sampling and dispensing motions of the reaction solution were performed using a syringe pump (C3000; TriContinent, Auburn, CA, USA) and a needle (200-mm G19; LK Lab, Namyangju, Republic of Korea). For the sampling action, the needle passed through the end-effector's hollow center, perforated the vial cap's septa (with a maximum of 30 N), and inhaled the solution with the retraction of the syringe piston. The solution was dispensed to another vial by the advancing motion of the syringe piston. The needle is moved by a linear motion mechanism, which includes a needle-motion guide, ball-screw, and needle-motor assembly.

Washing device
When each sampling step is completed, the associated components, including the needle, tubing, and syringe pump, should be cleaned in the washing device to avoid cross-contamination. The washing device has six washing columns of the same design for various applications, as shown in fig. S17. The needle was placed into one of the washing columns, which was filled with the washing solvent. The needle surface was cleaned using a flowing washing solvent. A new solvent was continuously introduced from the bottom of the column using a peristaltic pump (Reglo; Ismatec, Wertheim, Germany). The solvent overflowed to the top of the column and drained into waste. Meanwhile, the syringe pump of the end-effector infusion and withdrawal of the washing solvent repeatedly to clean the inside of the needle and the connecting tubing. In this study, dichloromethane, deionized water, and acetone were used sequentially in different washing columns to remove lipophilic and hydrophilic substances.

<u>Sample-preparation module</u>
The sample-prep. module shown in fig. S18 pretreats the sampled solution from the reaction module for LC-MS analysis and is based on a commercial MultiPurpose Sampler (MPS; Gerstel, Mülheim an der Ruhr, Germany) for dilution, mixing, filtration, and injection to LC-MS. The LC-MS separation mode primarily uses the reverse phase, which always contains water. The presence of water can lead to low solubility of organic electronic material, resulting in potential precipitation during sample injection. To prevent this, the sample is diluted 100-300 times with THF, which has high solubility for the sample. The process starts with dispensing 1980 μL THF into an empty sample vial and transferring it to the reaction module by the transfer robot. A small portion of the reaction solution (20–25 μL) is taken from a reaction vial and placed in a sample vial. The sample vial returns to the sample-prep. module and is shaken for mixing. Then, 850 μL of the solution is filtered through a 0.45-μm polytetrafluoroethylene (PTFE) filter (Whatman, Buckinghamshire, UK) to remove particles and diluted again with 1500 μL THF. Finally, 5 μL of the processed solution is injected into the LC-MS. To ensure proper mixing of the mobile phase and sample solution and reduce the risk of precipitation during injection, a 10 μL in-line mixer is implemented before the column selection valve. This enhancement improves the quantification reliability during the LC-MS analysis. To avoid cross-contamination, the liquid-handling devices in the MPS were cleaned with THF and isopropyl alcohol between each run.

<u>Analysis module</u>
The analysis module calculates the conversion yield of a chemical reaction using LC-MS (TSQ Quantis; Thermo Fisher Scientific, Waltham, MA) and sends it to the decision-making module to determine the direction of the experiment. It also provides additional information, such as the generation of side products and kinetics, to study the reaction mechanism. The conversion yield is defined as the number of molecules of the reference compound among the starting materials to be converted into the target compound and calculated from the ratio of the peak areas at 254 nm on the chromatogram, as described in eq. S1. This value does not indicate the absolute conversion yield because the light absorption coefficient varies for each compound based on concentration. Therefore, this concept is an indirect indicator to explore the optimal synthetic recipe for a particular reaction scheme.

$$\text{Conversion yield} = \frac{\text{Area of target compound}}{\sum \text{Area of (reference compound + target compound + byproducts)}} \quad , \text{(S1)}$$

where the reference compound refers to a species with a low equivalence ratio among the starting compounds.

In general laboratories, researchers determine the LC conditions after repeated experiments. However, the Synbot should provide the analysis results immediately, although the reaction conditions of each trial varies each time. To fulfill this requirement, we devised an alternate approach. First, five standard reverse-phase (RP) LC protocols are developed in advance, and the optimal protocol is automatically selected based on a decision tree. The standard LC protocols are summarized in table S5 and were developed so that the total analysis time was within 10 min. CORTECS C18 column (90 Å, 1.6 μm, 2.1 × 50 mm; Waters, Milford, MA) is used as a stationary phase and the flow rate is fixed at 0.3 mL/min in all protocols. Acetonitrile and 2-propanol were used as strong eluents to separate polar and non-polar substances, respectively. In addition, the protocols were refined by changing the ratio of the strong eluents over time. The pH affects the retention time and pKa of some chemicals; thus, it is necessary to use a buffer to suppress ions and maintain a stable pKa. For amine compounds widely used in electronic organic materials, a 10 mM acetate buffer (ammonium acetate + acetic acid, pH 4.7) was added.

A simple selection rule for the LC protocol was constructed, as explained in fig. S19. Acetonitrile-based eluents were used for organic compounds, while 2-propanol was added to the metal complexes. Each molecule is assumed to be further non-polar as its molecular weight increases. In the validation test with 78 substances (69 organic compounds and nine metal complexes), most substances were separated within the desired time. However, in some cases of bulk molecules with fused-ring cores, the target materials are not eluted from the column at a reasonable time, or poor peak shapes are observed because of their lower solubility. In this case, the protocol was slightly modified by increasing the ratio of strong solvents (e.g., acetonitrile and isopropyl alcohol) or changing the strong solvents to less polar solvents (e.g., THF). Nonetheless, the pre-defined protocols and selection rules continue to cover only a limited portion when considering the vast chemical space, and intermittent human intervention is required. Therefore, we plan to upgrade the analysis module to a closed-loop form that will optimize the protocol if the target molecule is determined to be outside the range of the existing model.

Transfer-robot module

The transfer-robot module transfers vials between modules, supplies new vials, and collects used vials. It is composed of an 8-m-long rail system (1-axis gantry; Festo), a robot arm (UR3e), and other supplementary components, such as the trays for reaction and sample vials and the QR code reader (DataMan 260; Cognex, Natick, MA) to track the vials, as presented in fig. S20. The robot arm moves on the rail to manipulate the vials using a modified Hand-E (Robotiq, Lévis,Canada) gripper. The rail system and robot arm were controlled by an EtherCAT master (PCIe-R0805-ECAT; Ajinextek, Daegu, Republic of Korea) connected to a servo drive (CMMT-AS-C4-3A-EC-S1; Festo) and TCP/IP client interfaces of Universal Robots (https://www.universal-robots.com/articles/ur/interface-communication/overview-of-client-interfaces/) on a remote computer, respectively.

Reliability of the Synbot

The reliability of the Synbot was verified in terms of experimental reproducibility. One of the most important reactions in organic electronic applications is the aromatic coupling between C-

C, C-N, or C-O. The coupling reactions use various types of catalytic groups and additives. Considering this, three representative reactions were selected, as summarized in table S6.

The dispensing performance and consistency of the kinetic curves were evaluated in 12 experiments (twice for each reaction slot) for each reaction. For the dispensing of powders, liquids, and solvents, the mean absolute errors (MAEs) were ≤ 0.73 mg, and the CVs were ≤ 2.55%. The CVs of the conversion yields with time were less than 5%. Moreover, when the conversion yields reached their maximum values, the CVs for Cases 1 to 3 decreased to 1.4, 1.7, and 2.5%, respectively, as summarized in table S7. As a result, the experimental reproducibility of the Synbot is within a reasonable range, considering its complexity in terms of workflow and equipment.

Case study of autonomous synthesis

Experimental procedure

Every experiment started with retrosynthesis and manual loading of the predicted chemicals to the pantry module. Common reagents are usually kept in the pantries, but case-specific reagents, including reactants, are prepared separately, depending on the situation. Then, autonomous synthesis to search for an optimal recipe begins within a predetermined chemical search space for each type of reaction. In principle, it is not necessary to set the chemical space in advance. However, it is a strategy used for an efficient search because there are specific catalysts or frequently used reagents depending on the reaction type. The limited space of the pantry module is another reason for this. The detailed chemical search space is explained in the following subsection.

The reaction types were determined by template-based matching. However, some of the coupling reactions between heteroatoms belong to different reaction types, such as the Buchwald amination and Ullmann reactions. In this case, Buchwald amination was chosen for C-N coupling, and the Ullmann reaction was selected for other C-hetero-atom couplings, such as C-O and C-S. Then, the MPNN models proposed three recipes for initial trials, and the remaining three conditions were randomly selected by the MLHS as an exploration strategy (as previously explained in the "DoE and optimization" subsection). The optimization process continues under the supervision of the decision-making module.

Search space for synthetic recipes

Frequently used reagents were extracted from the Reaxys DB to define the search space according to the reaction type. However, because of the possibility of not finding a suitable recipe in this range, the search space has been somewhat extended to a slightly low-use reagent. The search space was defined in terms of the catalyst, ligand, base, and solvent, as summarized in table S8.

Catalysts are divided into three categories, as shown in table S9: Pd-type1, Pd-type2, and Cu-catalysts. Most palladium catalytic cycles start with the active catalytic $L_nPd(0)$ species, where L_n represents the ligands to stabilize Pd(0) complexes. $Pd(PPh_3)_4$, a widely used catalyst in Suzuki coupling, can be used in its Pd(0) form without additional ligands. However, some catalyst sources, such as palladium acetate, require additional ligands for activity. The preference for the catalysts varies marginally depending on the type of reaction. Thus, the search space of palladium catalysts is further split into two subcategories: "Pd-type1" which includes $Pd(PPh_3)_4$ as a main species, and "Pd-type2" where catalysts other than $Pd(PPh_3)_4$ are preferred. In the case

of Sonogashira coupling reaction, copper iodide is always added to the reaction mixture, so the actual search space is “Pd-type1.”

The search space for phosphate ligands to activate palladium catalysts is classified according to their chemical characteristics: monodentate phosphate ligands, bidentate phosphine ligands, and bulky electron-rich dialkylbiaryl phosphines (table S10). The ligand search space for the copper catalysts is also defined in table S11.

Inorganic bases are usually used in catalytic coupling reactions, which we focus on to maintain the catalytic activity during the reaction. To set the search space, counter anions of the inorganic bases with pKa values between 3 and 14 were considered. The most frequently used inorganic bases were chosen for each counter anion, as listed in table S12.

Reaction media are generally pure organic solvents for the synthesis of organic electronic materials, but mixed solvents, including water, are widely used for Suzuki coupling reactions. To consider both cases, we define pure organic (“Solvent-org.”), and water-soluble (“Solvent-aq.”) solvents together. To limit the number of cases, the mixed solvent is treated as a single element, and the entire search space of the solvent is designated in table S13. The reaction temperature was determined based on the boiling point of the solvents at intervals of 10 °C. The exceptions were dimethyl sulfoxide (DMSO) and *o*-xylene. The high-temperature reaction of DMSO may cause an unexpected side reaction due to methylation, and thus the reaction temperature was set to 110 °C even though the boiling temperature was 189 °C. Because *o*-xylene was used as an alternative to toluene at a marginally higher temperature, its reaction temperature was set to 120 ^{o}C, which is 10 ^{o}C higher than toluene. The reaction temperatures of the mixed solvents were determined by referring to the average boiling points of the solvents.

Summary of the characteristics of the Synbot

Advanced recipe optimization

It employs a hybrid-type dynamic optimization approach for recipe optimization. This algorithm, based on message-passing neural networks and Bayesian optimization, demonstrates exceptional efficiency. In our research (*34*), we extensively validated various optimization algorithms and compared their performance with that of chemists in rapidly finding optimal recipes.

Experiment representation

Within the system, researchers only input information about a target molecule and yield, then AI selects an experimental recipe with a chemical representation, converting it into a task for an automated experimental robot. We have defined the experiment recipes and robot tasks, facilitating a wide range of experiments.

Reliability of the system

The Synbot is designed for securing high reliability and repeatability. The usage of tubes and pumping systems has been minimized to mitigate clogging and cross-contamination issues typical in repetitive experiments. Instead, a disposable vial-based batch-type reaction configuration is adopted.

The dispensing module operates in a nitrogen environment to improve reagent stability. However, we encountered challenges related to static electricity generation, affecting the precision of dispensing. This issue was resolved by implementing an ionizer and modifying the

process flow. And a dedicated syringe-based dispenser was also developed to handle small liquid volume.

And a stable vial-cap structure with refluxing function is devised to guarantee chemical durability during the long thermal and mechanical cycles.

Automation of analysis

Unlike other systems that limit analytical targets or require human intervention, our system features an LC-MS protocol selection model based on product's chemical characteristics. This enables not only the automatic operation of the system but also the continuous monitoring of reaction-status.

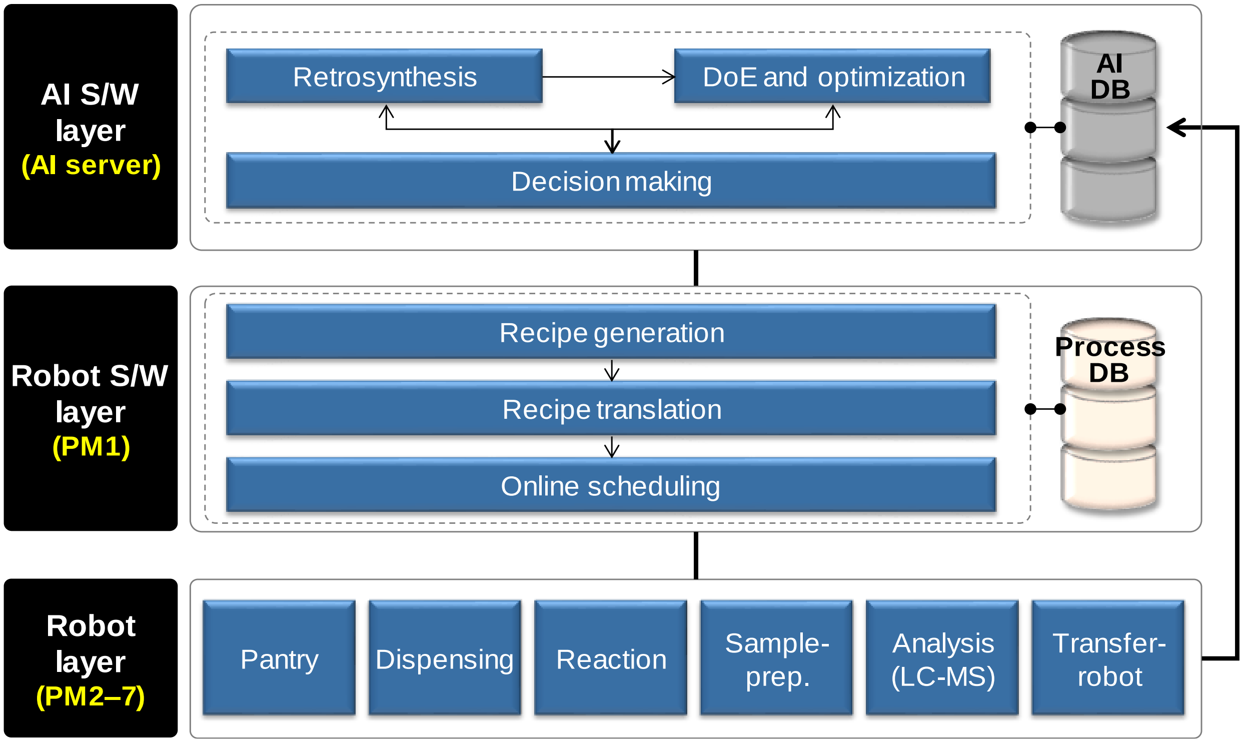

Fig. S1. Architecture of the Synbot.

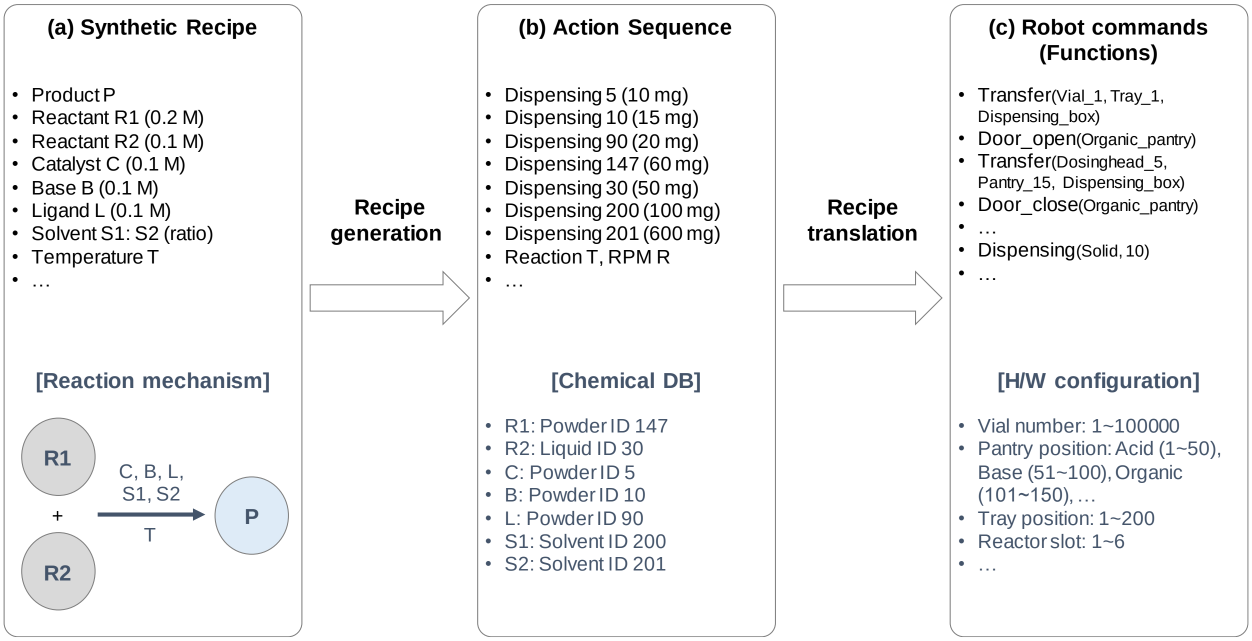

Fig. S2. Working mechanism of recipe generation and translation modules.

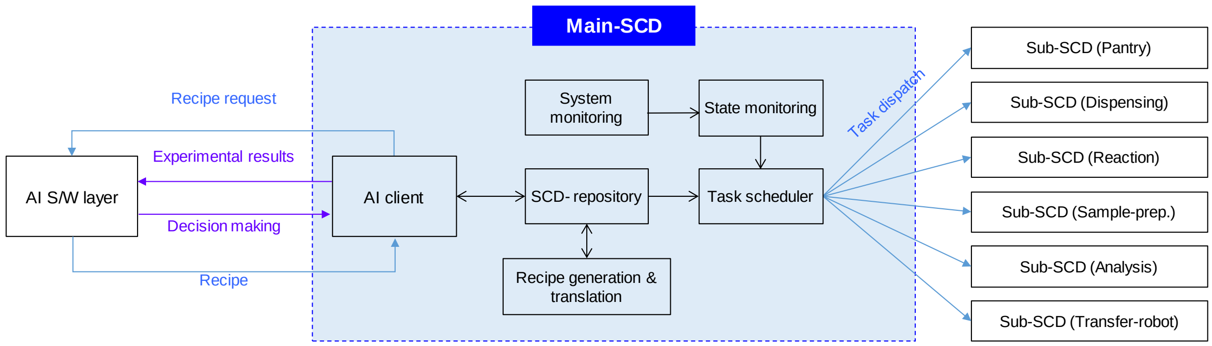

Fig. S3. Working procedure of the online scheduling.

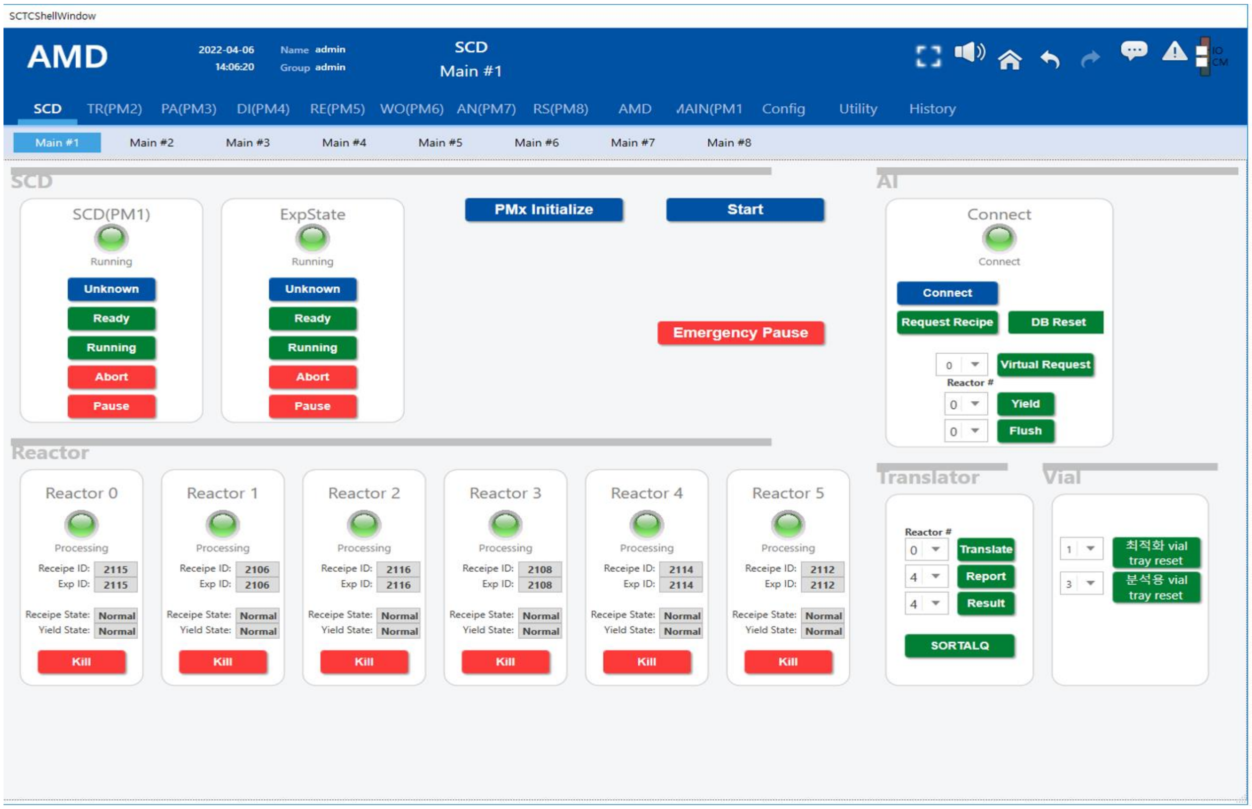

Fig. S4. Master GUI of the Synbot.

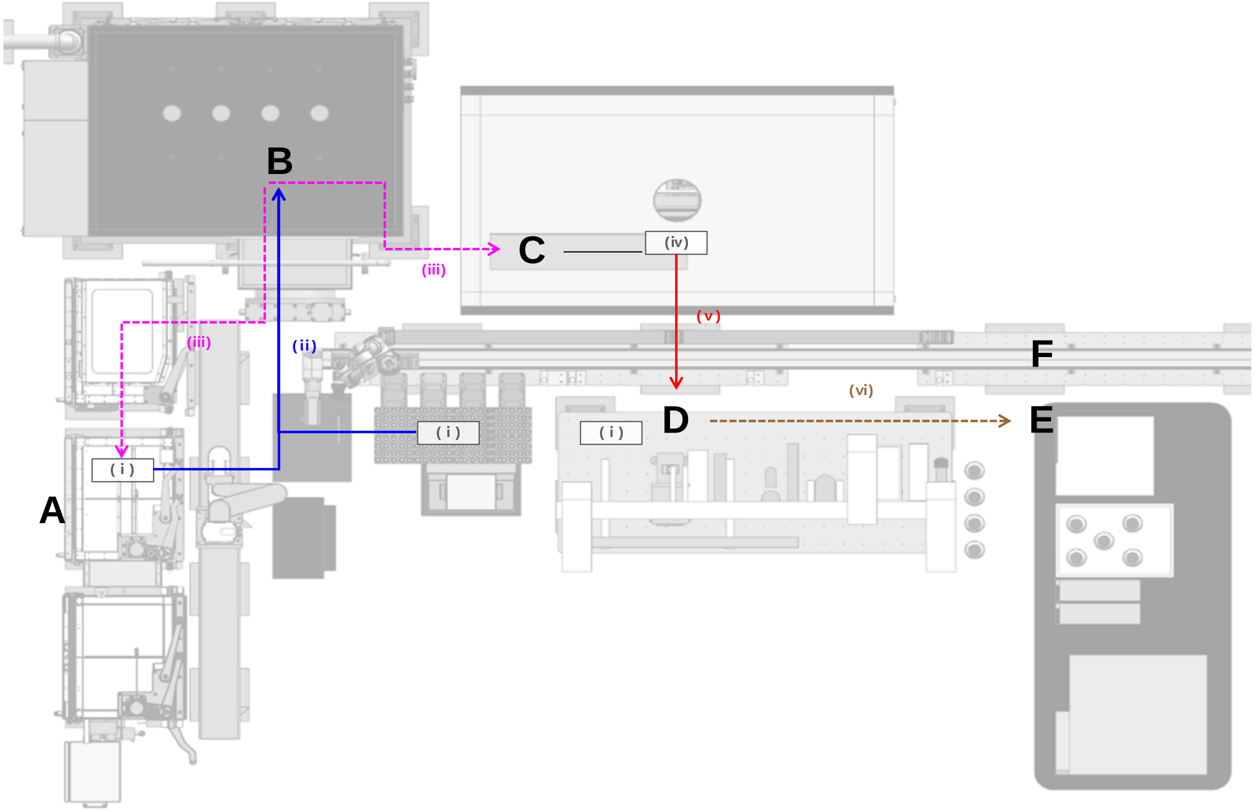

Fig. S5. Experimental workflow of the Synbot. (**A**) Pantry module. (**B**) Dispensing module. (**C**) Reaction module. (**D**) Sample-prep. module. (**E**) Analysis module. (**F**) Transfer-robot module.

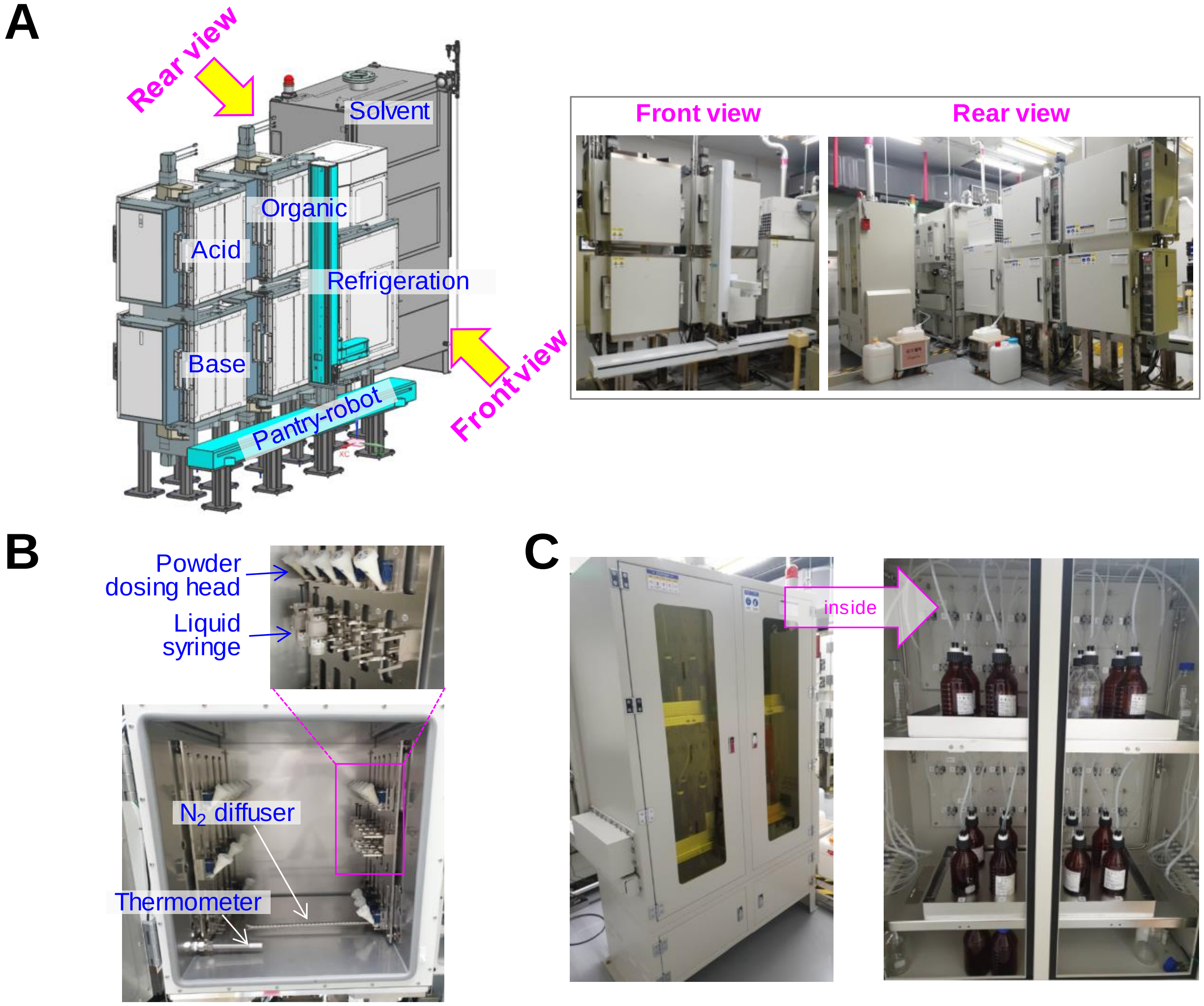

Fig. S6. Pantry module. (**A**) Schematic and pictures. (**B**) Inside of the acid pantry. (**C**) Solvent pantry.

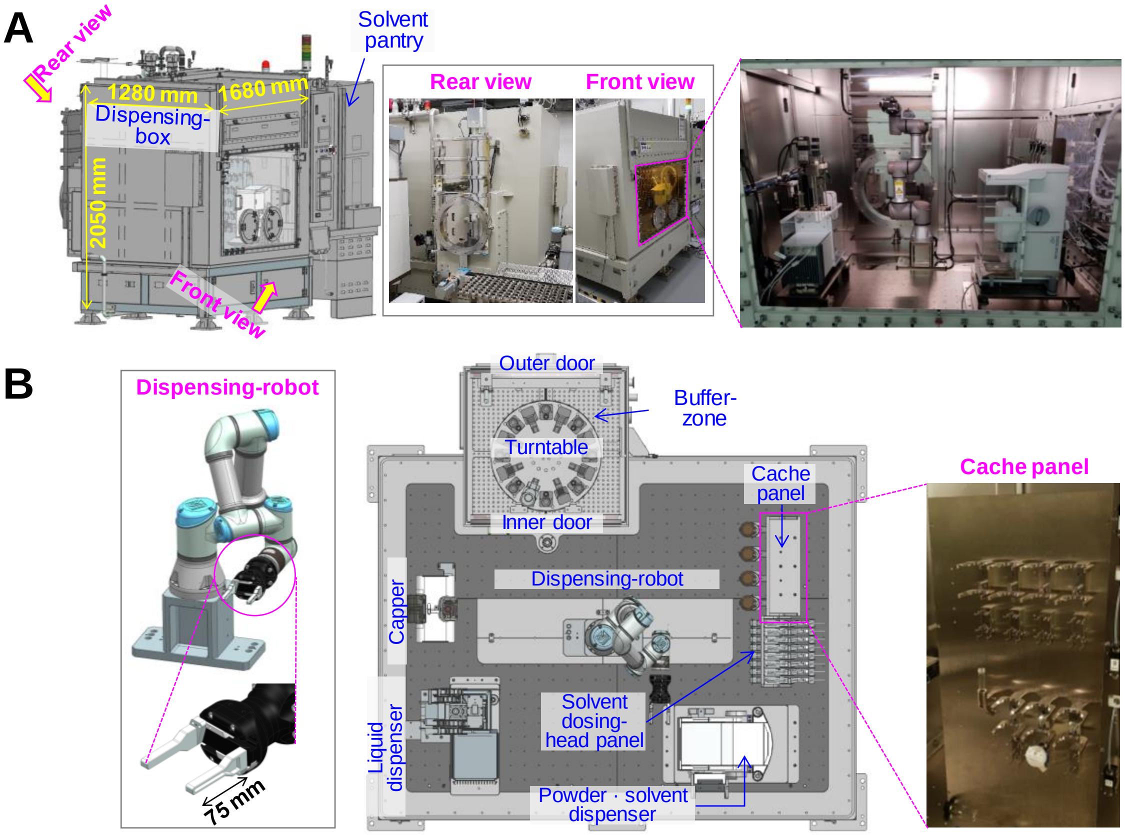

Fig. S7. Dispensing module. (**A**) Schematic and pictures. (**B**) Top view of module interior.

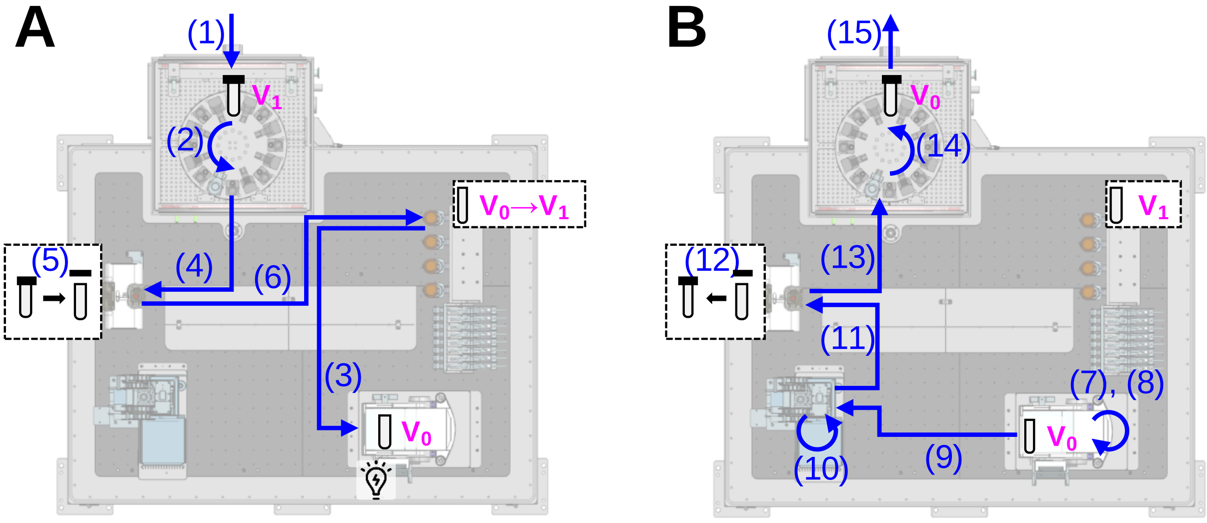

Fig. S8. Dispensing procedure. (**A**) Preparation. (**B**) Dispensing and post-processing.

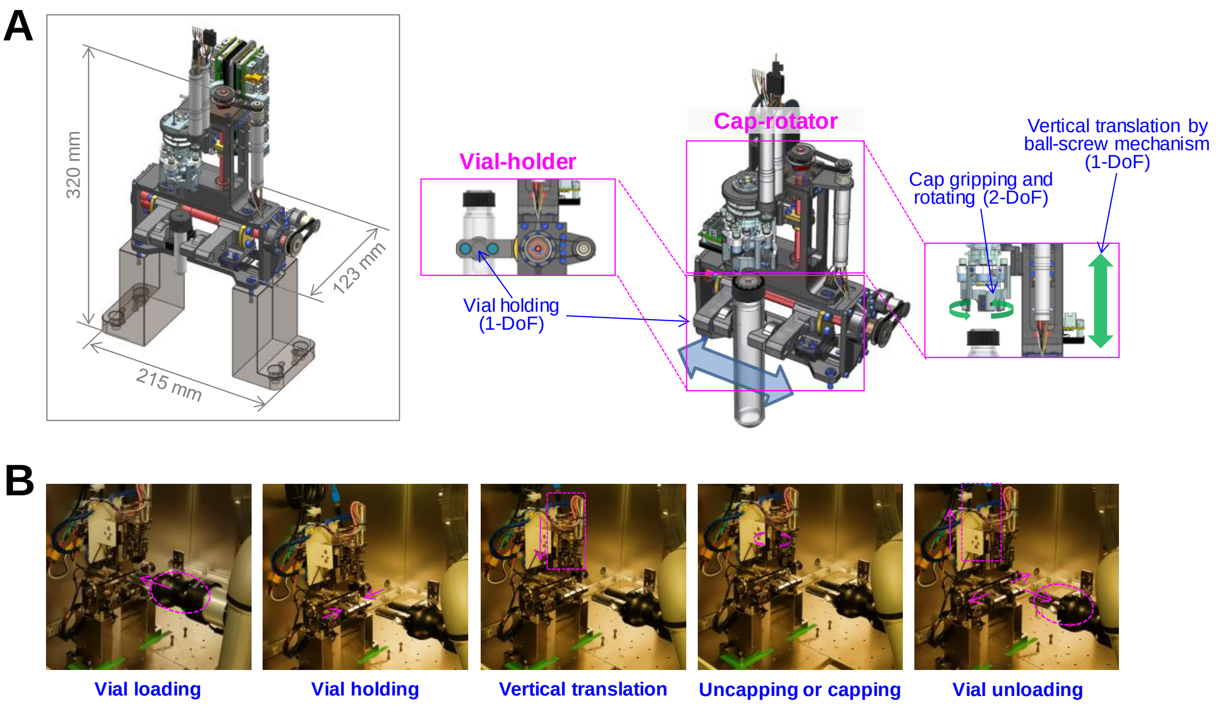

Fig. S9. Capper. (**A**) Schematics. (**B**) Uncapping and capping processes.

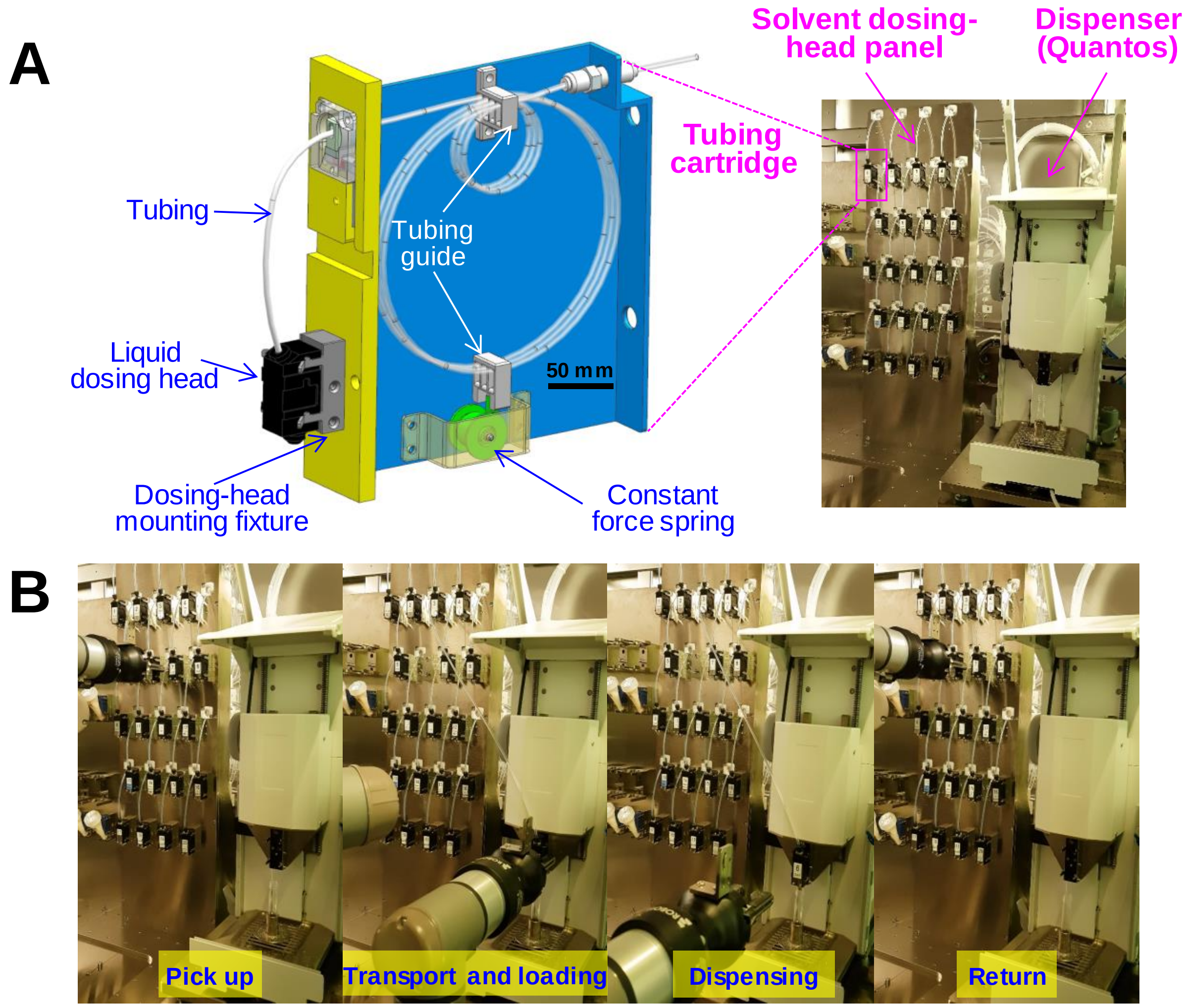

Fig. S10. Handling of liquid dosing heads. (**A**) Solvent dosing-head panel and tubing cartridge. (**B**) Loading process of a liquid dosing head into the dispenser.

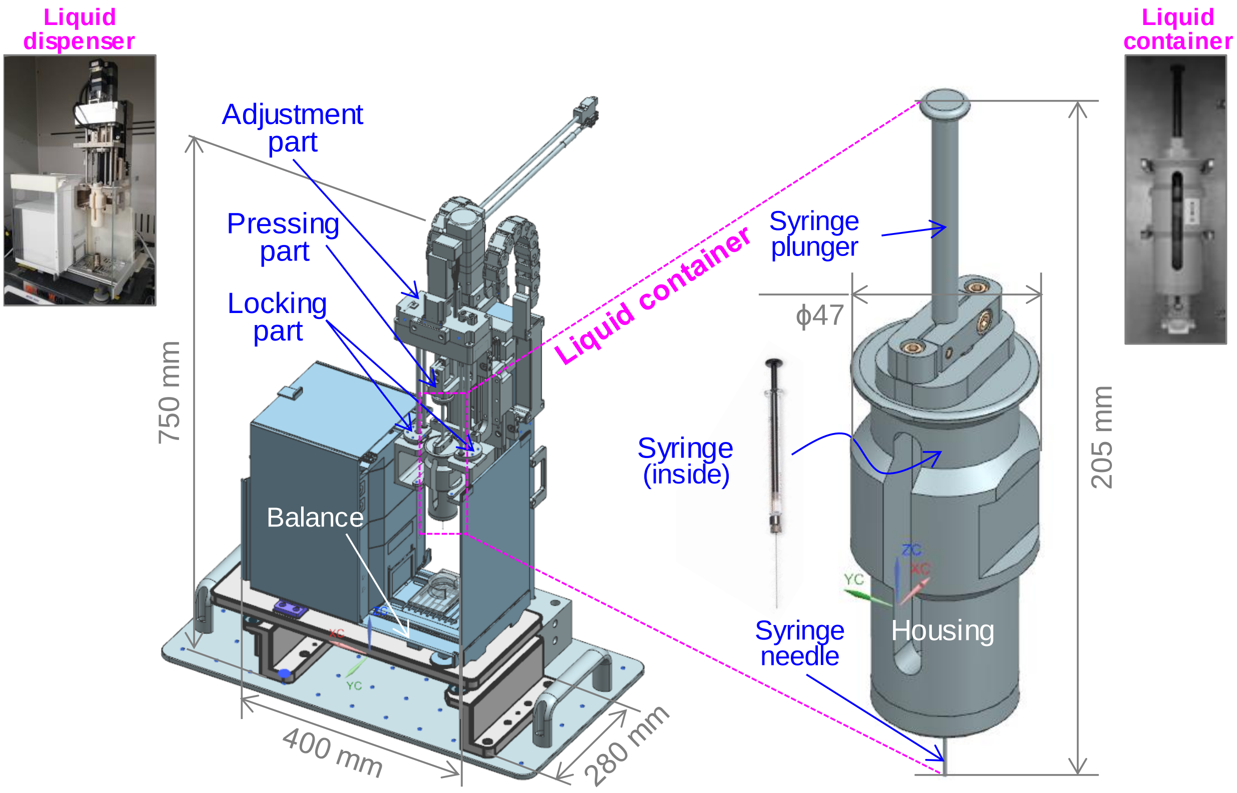

Fig. S11. Liquid dispenser and container.

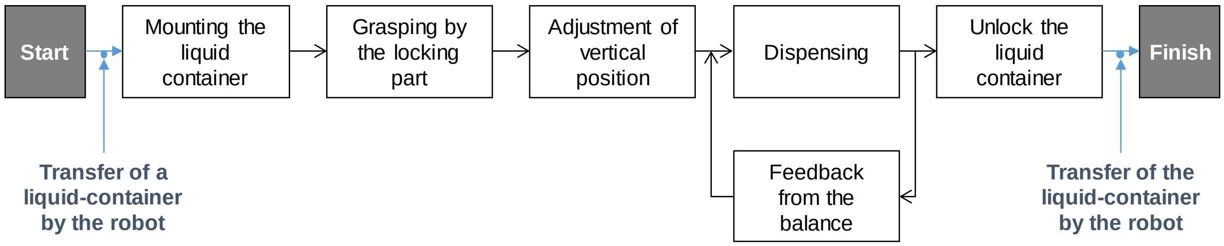

Fig. S12. Sequence of liquid dispensing.

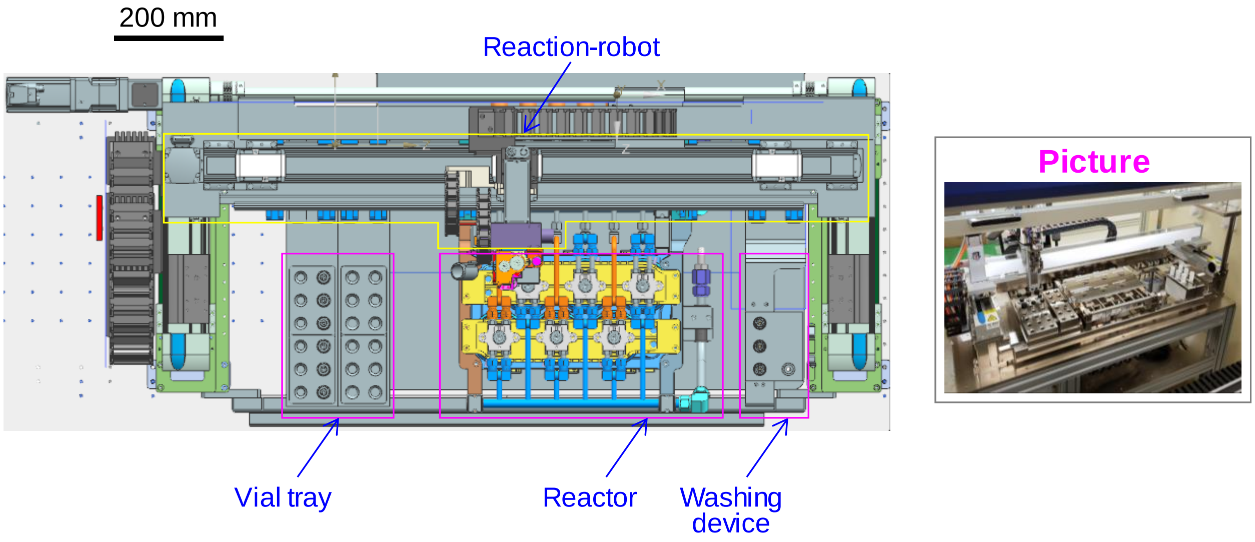

Fig. S13. Reaction module.

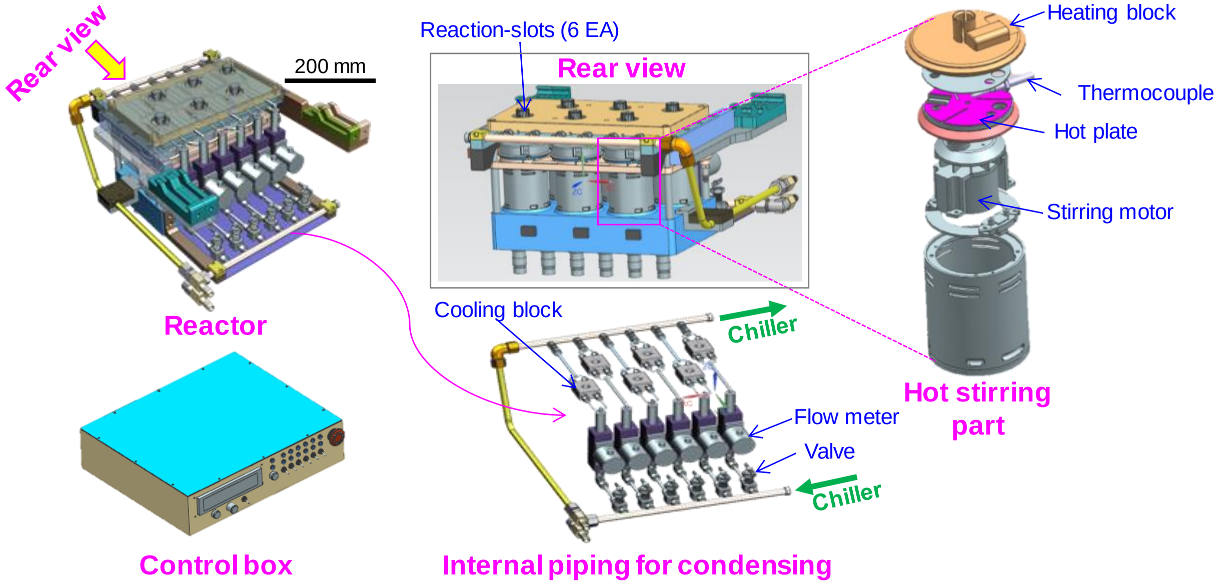

Fig. S14. Reactor.

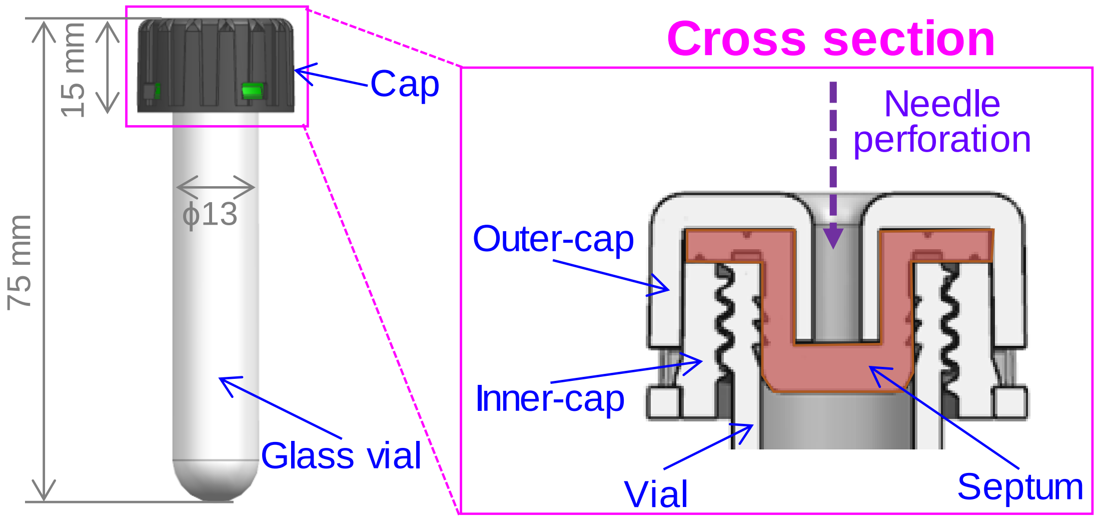

Fig. S15. Reaction vial.

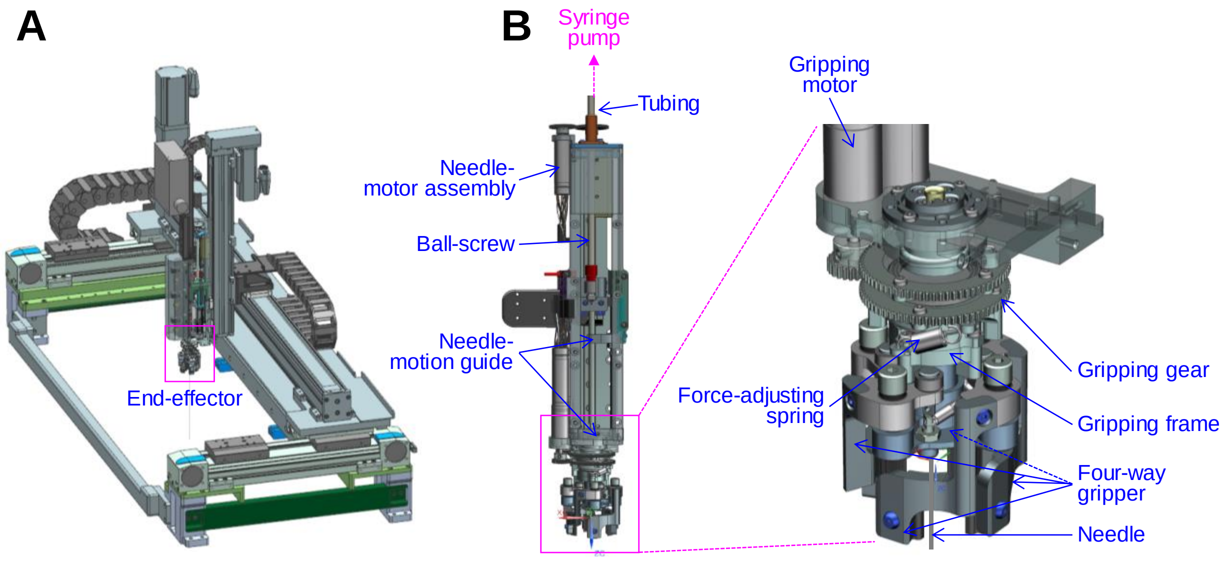

Fig. S16. Reaction robot. (**A**) XYZ-stage robot. (**B**) End-effector.

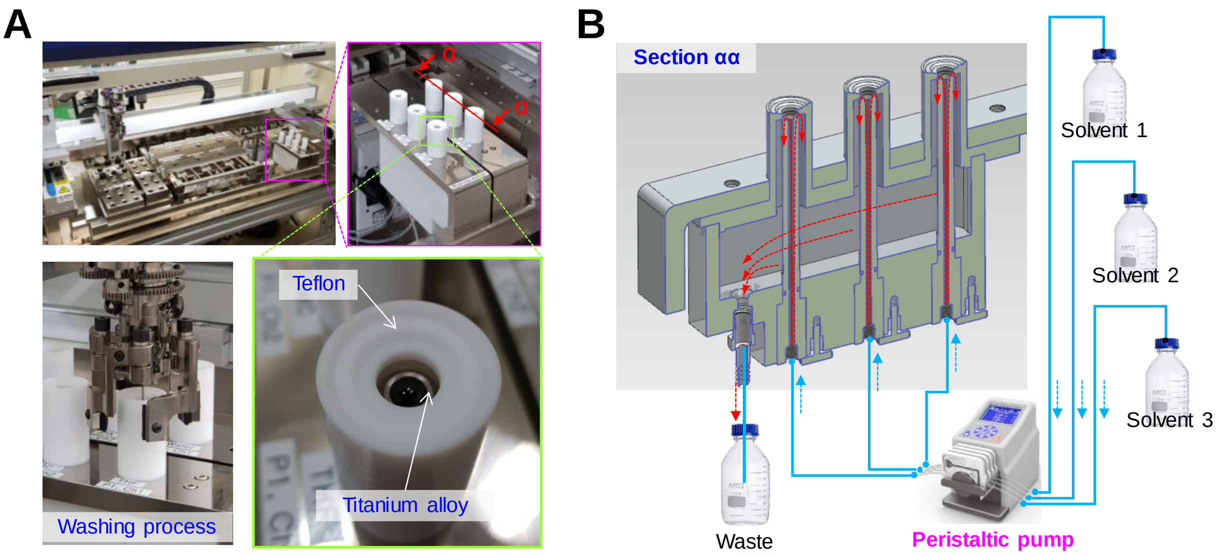

Fig. S17. Washing device. (**A**) Pictures of structure and process. (**B**) Working mechanism.

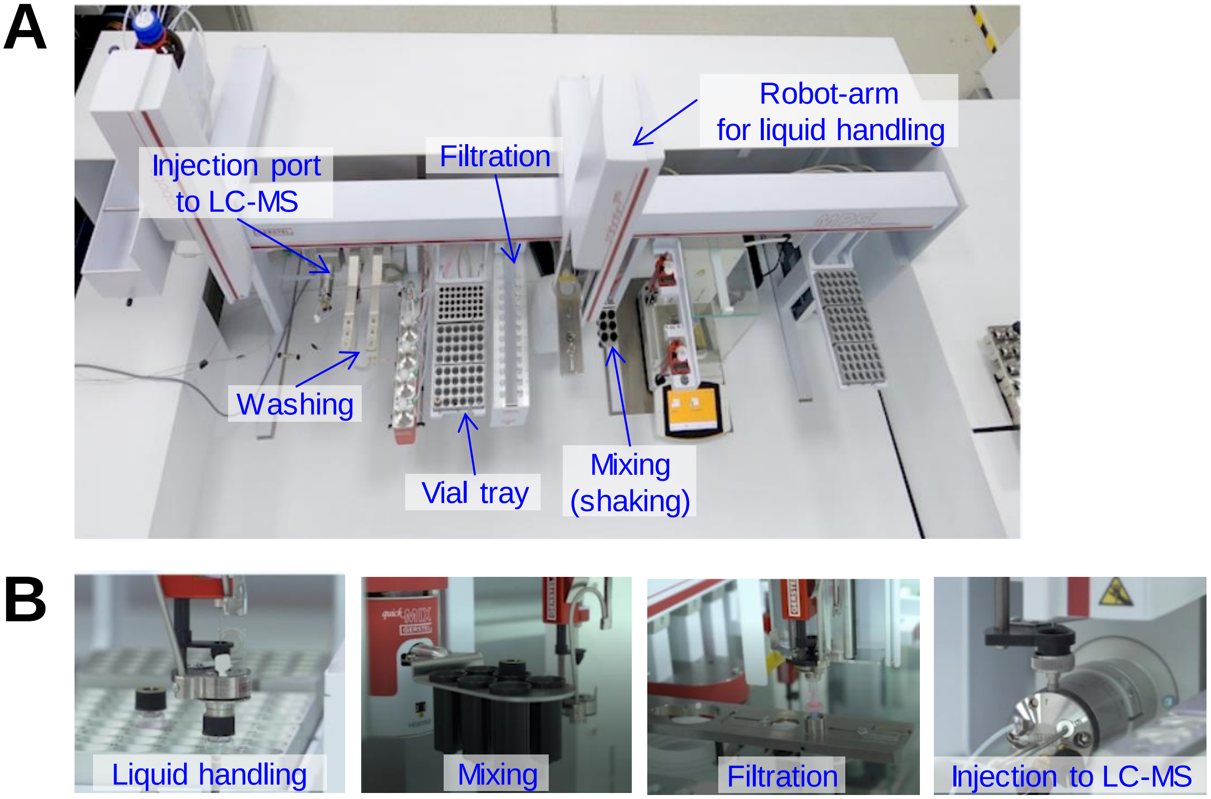

Fig. S18. Sample-preparation module. (**A**) Module layout. (**B**) Unit processes.

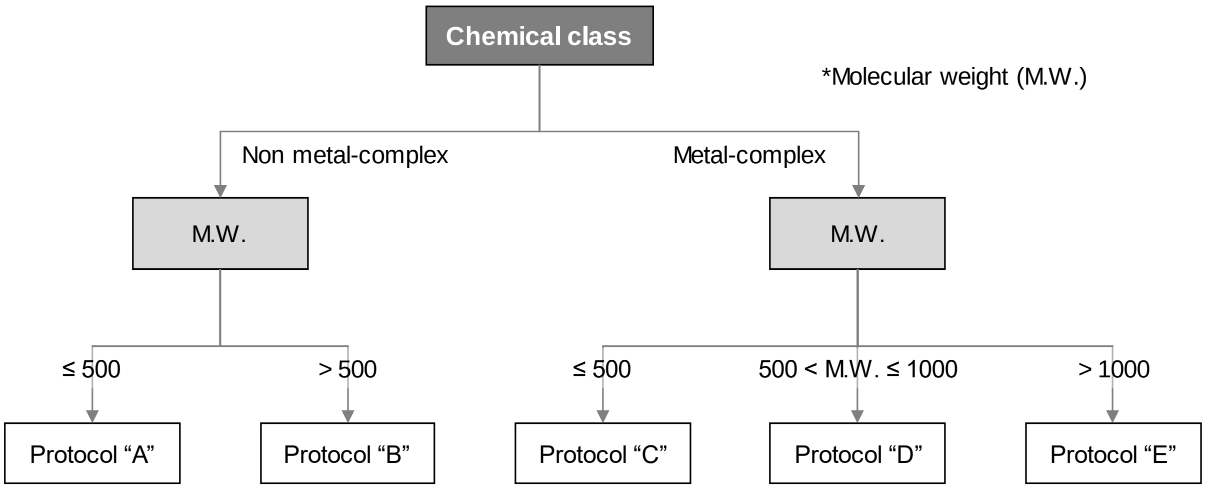

Fig. S19. Selection rule of liquid chromatography protocol.

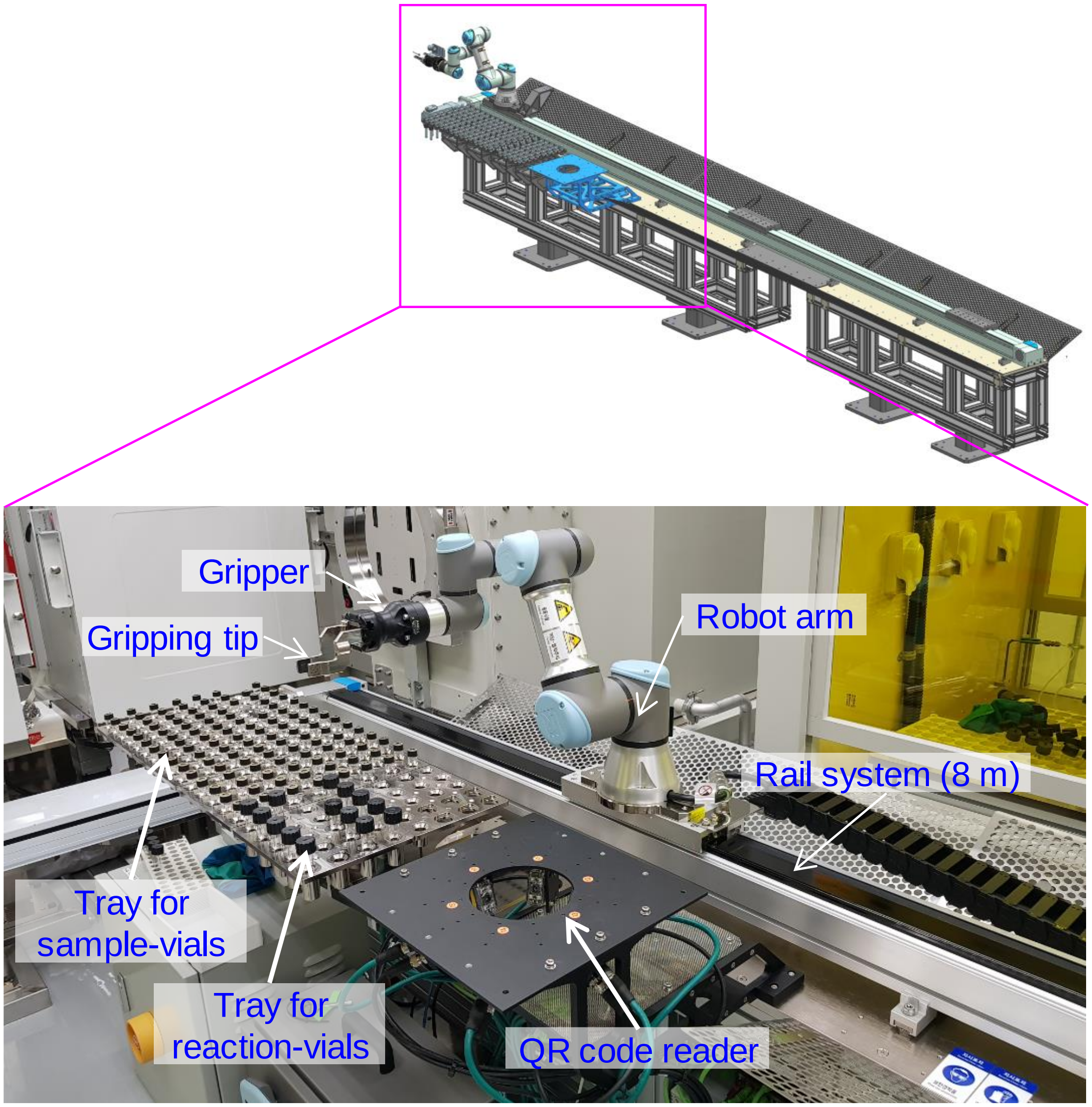

Fig. S20. Transfer-robot module.

Table S1. Performance of retrosynthesis models.

Rank	Template-based model	Tied-two-way transformer	Ensemble model
Top-1	67.5%	70.0%	74.5%
Top-2	70.4%	79.3%	84.6%
Top-3	71.4%	82.3%	86.1%
Top-4	72.0%	83.8%	87.1%
Top-5	72.4%	84.7%	88.0%
Top-6	72.6%	85.2%	88.6%
Top-7	72.8%	85.5%	89.2%
Top-8	73.0%	85.8%	89.7%
Top-9	73.1%	85.9%	90.1%
Top-10	73.2%	86.0%	90.5%

Table S2. Tasks of the Synbot.

Task	Description
Supply of vial and dosing heads	Provision of a new vial and dosing heads to the dispensing module as a preparation step of dispensing.
Dispensing	Dispensing process of chemicals into a reaction vial including uncapping and capping of vial cap.
Reaction	Transfer of the dispensed vial to the reaction module and conducting chemical reaction.
Analysis	Sampling of reaction solution, and LC-MS analysis after sample preparation.

Table S3. Specification of the pantries.

Pantry	Inner size (W×D×H, mm)	Storage capacity	Atmosphere	Temperature (^{o}C)
Acid	560 × 530 × 580	Solid 25 EA<br>Liquid 5 EA	Nitrogen (≥ 99% v/v)	Room temperature
Base		Solid 25 EA<br>Liquid 5 EA		
Organic		Solid 50 EA<br>Liquid 10 EA		
Refrigeration		Solid 20 EA<br>Liquid 10 EA	Air	≤ 4
Solvent	1227 × 1196 × 586	Solvent 20 EA	Air	Room temperature

Table S4. Coolant flow rate and temperature of the condensing blocks.

Slot number	1	2	3	4	5	6
Flow rate (L/min)	3.3	3.3	3.3	3.3	3.3	3.3
Condensing block temperature (℃)	11.6	11.8	11.2	11.7	11.8	12.1

Table S5. Standard protocols for reverse-phase liquid chromatography.

Protocol	Elution solvent		
	i. Weak eluent	ii. Strong eluent	Ratio (ii/(i+ii), %)
A	Water	Acetonitrile	10 → 70 (@ 2 min) → 95 (@ 5 min)
B	Water	Acetonitrile	70 → 95 (@ 5 min)
C	Water + Acetonitrile*	Acetonitrile + 2-Propanol**	5 → 50 (@ 5 min)
D	Water + Acetonitrile*	Acetonitrile + 2-Propanol**	5 → 20 (@ 1 min) → 95 (@ 5 min)
E	Water + Acetonitrile*	Acetonitrile + 2-Propanol**	5 → 70 (@ 1 min) → 95 (@ 5 min)

*Water:Acetonitrile = 2:3, **Acetonitrile:2-Propanol = 1:10

Table S6. Reaction schemes for the reliability test of the Synbot.

		Case 1	Case 2	Case 3
Target material		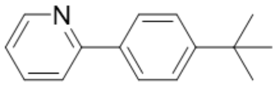	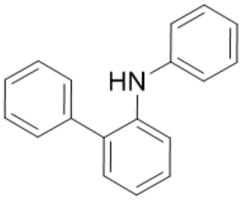	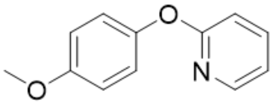
Reaction mechanism		Suzuki coupling	Buchwald reaction	Ullmann reaction
Reagents	Reactants	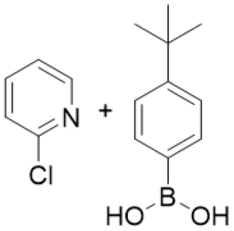	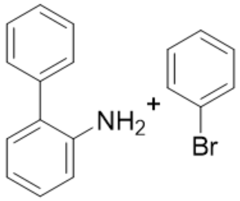	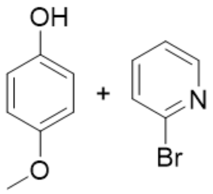
	Catalyst	$Pd(PPh_3)_4$	$Pd_2(dba)_3$	CuI
	Ligand	–	SPhos	1,10-Phenanthroline
	Base	K_2CO_3	K_2CO_3	K_2CO_3
	Solvent	THF:H_2O (3:1)	THF	Dimethylformamide
Reaction condition	Concentration (M)	0.2	0.2	0.2
	Temperature (℃)	80	80	100
Analysis time (h)		1, 3, 6	1, 3, 6, 9	1, 3, 6, 9, 12

Table S7. Results of the reliability test.

(Case 1)	Dispensing accuracy (mg)						Conversion yield (%)		
	Reactant 1 (Liquid)	Reactant 2 (Solid)	Catalyst (Solid)	Base (Solid)	Solvent 1	Solvent 2	1 h	3 h	6 h
Target	22.71	42.73	11.56	82.92	654.13	244.53	–	–	–
Trial 1	22.36	42.40	11.32	83.08	654.11	244.62	88.3	94.9	98.1
Trial 2	22.43	42.34	11.43	82.42	654.22	244.68	83.4	95.3	98.9
Trial 3	22.48	42.33	11.47	82.68	654.06	244.67	87.5	94.8	97.9
Trial 4	22.39	42.36	11.52	82.79	654.01	244.69	78.1	93.3	98.0
Trial 5	22.03	42.40	11.67	83.76	654.21	244.61	89.2	97.5	100.0
Trial 6	22.49	42.45	11.39	82.36	654.32	244.68	83.7	92.4	97.9
Trial 7	22.61	42.42	11.49	83.26	654.20	244.63	87.8	97.1	99.9
Trial 8	22.44	42.39	11.46	82.48	653.98	244.65	88.1	95.9	98.2
Trial 9	22.12	42.47	11.62	82.93	653.87	244.80	78.5	91.0	95.2
Trial 10	22.31	42.36	11.25	82.82	653.75	244.58	83.5	94.2	97.3
Trial 11	21.90	42.39	11.39	86.23	653.82	244.66	81.3	93.3	96.9
Trial 12	21.98	42.39	11.48	82.21	653.51	244.60	80.7	93.1	96.6
Ave.	22.30	42.39	11.46	83.09	654.01	244.66	84.2	94.4	97.9
S.D.	0.23	0.04	0.12	1.08	0.23	0.06	4.0	1.9	1.3
MAE	0.42	0.34	0.13	0.61	0.20	0.13	–	–	–
CV (%)	1.03	0.10	1.02	1.30	0.04	0.02	4.7	2.0	1.4

(Case 2)	Dispensing accuracy (mg)						Conversion yield (%)			
	Reactant 1 (Solid)	Reactant 2 (Liquid)	Catalyst (Solid)	Ligand (Solid)	Base (Solid)	Solvent	1 h	3 h	6 h	9 h
Target	33.85	34.54	18.31	16.42	82.92	868.53	–	–	–	–
Trial 1	36.08	34.64	18.63	16.66	83.65	868.63	40.3	69.8	85.4	91.4
Trial 2	34.20	35.16	18.71	16.73	84.28	868.65	40.4	70.6	86.7	93.6
Trial 3	33.97	34.71	18.57	16.7	82.99	868.72	41.3	70.2	86.7	93.0
Trial 4	34.02	33.36	18.53	16.86	83.06	868.52	41.5	71.6	86.3	91.4
Trial 5	34.15	33.36	18.57	16.77	83.84	868.75	41.6	70.8	86.5	92.5
Trial 6	33.85	33.19	18.31	16.52	83.83	868.54	42.0	70.9	85.7	91.3
Trial 7	35.60	33.84	18.67	16.67	83.03	868.47	40.9	69.5	84.4	91.1
Trial 8	33.99	33.82	18.47	16.52	83.07	868.59	43.1	70.0	85.8	91.6
Trial 9	33.89	33.81	18.48	16.63	83.27	868.66	42.5	71.1	86.2	91.8
Trial 10	34.22	33.80	19.02	16.74	83.26	868.67	40.7	68.4	81.3	87.5
Trial 11	34.18	34.21	18.53	16.48	82.91	868.21	44.5	74.0	87.2	91.9
Trial 12	34.62	33.61	18.74	16.79	83.41	868.56	41.8	70.2	84.1	90.2
Ave.	34.40	33.96	18.60	16.67	83.38	868.58	41.7	70.6	85.5	91.4
S.D.	0.71	0.61	0.18	0.12	0.43	0.14	1.2	1.4	1.6	1.5
MAE	0.55	0.73	0.29	0.25	0.46	0.12	–	–	–	–
CV (%)	2.07	1.79	0.95	0.71	0.51	0.02	2.9	1.9	1.9	1.7

(Case 3)	Dispensing accuracy (mg)						Conversion yield (%)				
	Reactant 1 (Liquid)	Reactant 2 (Solid)	Catalyst (Solid)	Ligand (Solid)	Base (Solid)	Solvent	1 h	3 h	6 h	9 h	12 h
Target	31.60	24.83	11.43	21.63	82.92	926.00	–	–	–	–	–
Trial 1	30.31	25.05	11.92	21.87	83.08	926.13	46.4	62.7	72.0	78.4	80.8
Trial 2	32.07	25.06	11.98	22.02	82.89	926.11	45.9	63.4	74.6	77.5	81.0
Trial 3	31.79	25.14	11.59	21.69	82.95	926.11	48.8	63.9	74.8	79.0	81.8
Trial 4	31.87	25.03	11.82	21.91	83.49	926.26	48.9	65.8	76.2	79.5	81.7
Trial 5	30.31	25.39	12.06	21.90	83.65	926.26	47.9	66.8	79.8	84.1	84.2
Trial 6	30.04	26.68	11.74	21.82	83.22	926.24	49.6	69.0	80.5	84.9	86.3
Trial 7	31.83	24.85	12.72	21.67	83.97	926.22	50.2	70.9	78.9	83.9	85.6
Trial 8	31.83	24.88	11.62	21.81	82.94	926.18	48.2	65.6	77.3	80.7	82.3
Trial 9	30.98	25.10	12.13	21.89	84.18	926.25	49.0	65.8	77.8	81.8	84.1
Trial 10	31.90	25.56	11.70	21.69	83.87	926.52	47.5	64.9	76.8	81.4	84.6

Trial 11	31.60	25.49	11.90	22.04	83.42	926.26	46.2	67.2	80.2	84.4	86.9
Trial 12	31.78	25.41	12.08	22.07	83.44	926.26	44.0	61.8	73.4	78.6	81.9
Ave.	31.36	25.30	11.94	21.87	83.43	926.23	47.7	65.7	76.9	81.2	83.4
S.D.	0.74	0.49	0.30	0.14	0.43	0.11	1.8	2.6	2.8	2.6	2.1
MAE	0.55	0.47	0.51	0.24	0.51	0.23	–	–	–	–	–
CV (%)	2.36	1.95	2.55	0.62	0.51	0.01	3.8	4.0	3.6	3.2	2.5
Ave. (average), S.D. (standard deviation)											

Table S8. Search space of each reaction type.

Reaction type	Catalyst	Ligand	Base	Solvent
Suzuki coupling	Pd-type1	Pd-ligand	Base-inorganic	Solvent-aq.
Still coupling	Pd-type1	Pd-ligand	Base-inorganic	Solvent-org.
Sonogashira coupling	Pd-type1 (+ CuI)	Pd-ligand	Base-inorganic	Solvent-org.
Heck	Pd-type1	Pd-ligand	Base-inorganic	Solvent-org.
Ullmann	Cu-catalyst	Cu-ligand	Base-inorganic	Solvent-org.
Buchwald Hartwig	Pd-type2	Pd-ligand	Base-inorganic	Solvent-org.

Table S9. Search space of catalyst.

Pd-type1	Pd-type2	Cu-catalyst
$Pd(PPh_3)_4$	$Pd(OAc)_2$	CuI
$Pd(OAc)_2$	$Pd(dba)_2$	Copper (pure)
$Pd(dba)_2$	$Pd_2(dba)_3$	Cu_2O
$Pd_2(dba)_3$	–	CuO
$PdCl_2$	–	$Cu(OAc)_2$
–	–	$CuSO_4$

Table S10. Search space of ligand for Pd-catalysts (Pd-ligand).

Ligand types	Pd-ligand candidates
Monodentate phosphate	Triphenylphosphine (PPh_3)
	Tri-tert-butyl phosphine ($PtBu_3$)
	Tricyclohexylphosphine (PCy_3)
	APhos
	Tri(o-tolyl)phosphine ($P(o\text{-}tol)_3$)
	Trifuran-2-yl-phosphine (TPF)
Bidentate phosphine	Xantphos
	DPEPhos
	BINAP
	DPPF
	DPPP
Bulky electron-rich dialkylbiaryl phosphine	SPhos
	XPhos
	tBuXphos
	BrettPhos
	DavePhos
	RuPhos
	JohnPhos
	CyJohnPhos

Table S11. Search space of ligand for Cu-catalysts (Cu-ligand).

Ligand types	Cu-ligand candidates
N-O type	DL-Proline
	2-Picolinic acid
	Dipivaloylmethane
	8-Quinolinol
	Dimethylglycine
	Sarcosine
Alkylamine	N,N-Dimethylethylenediamine (DMEDA)
	N-Tetramethylethylenediamine (TMEDA)
	(PMDETA)
	CyDA
	DMCyDA
	Cyclam
Biazaaryl	2,2'-Bipyridine (bpy)
	2,2'-Bi(γ-picoline)
	Tris(2-pyridylmethyl)amine (TPMA)
	1,10-Phenanthroline
	4,7-Dimethoxypenanthroline
	3,4,7,8-Tetramethylphenanthroline

Table S12. Search space of base (Base-inorganic).

Counter anion	Base-inorganic candidates
F^-	CsF
OAc^-	KOAc
HCO_3^-	$NaHCO_3$
CO_3^-	Na_2CO_3
	K_2CO_3
	Cs_2CO_3
PO_4^-	K_3PO_4
OH^-	NaOH
OEt^-	–
$OtBu^-$	NaOtBu

Table S13. Search space of solvent.

Solvent-org.		Solvent-aq.	
Candidates	Reaction temperature (℃)	Candidates	Reaction temperature
Toluene	110	1,4-Dioxane + Water (3:1)	100 ºC
1,4-Dioxane	100	THF + Water (3:1)	80 ºC
1,2-Dimethoxyethane (DME)	80	Toluene + Ethanol + Water (3:1:1)	100 ºC
THF	70	DMF	150 ºC
Dimethylforamide (DMF)	150	–	–
DMSO	110	–	–
Acetonitrile (MeCN)	80	–	–
o-Xylene	120	–	–

Table S14. Autonomous synthetic conditions for M1-1.

Trial number	Catalyst	Ligand	Base	Solvent	Reaction temperature (℃)	Maximum conversion yield
Reference for M1-3	$Pd(PPh_3)_4$	–	K_2CO_3	Toluene + Ethanol + Water (3:1:1)	120	86.5% @18.0 h
1	$Pd(PPh_3)_4$	–	K_2CO_3	THF + Water (3:1)	80	100.0% @ 1.0 h
2	$Pd(PPh_3)_4$	–	K_2CO_3	DMF	150	100.0% @ 3.5 h
3	$Pd(PPh_3)_4$	–	K_2CO_3	Toluene + Ethanol + Water (3:1:1)	100	100.0% @ 1.2 h
4	$Pd(OAc)_2$	SPhos	K_3PO_4	Toluene + Ethanol + Water (3:1:1)	100	100.0% @ 6.5 h
5	$PdCl_2$	DPPF	$NaHCO_3$	DMF	150	100.0% @ 1.0 h
6	$Pd(OAc)_2$	tBuXphos	K_2CO_3	Toluene + Ethanol + Water (3:1:1)	100	Not reacted

* Size of search space = 2,722 = (4 catalysts × 19 ligands + 1 catalyst without ligand) × 9 bases × 4 solvents

Table S15. Autonomous synthetic conditions for M1-3.

Trial number	Catalyst	Ligand	Base	Solvent	Reaction temperature (℃)	Maximum conversion yield
Reference	$Pd(PPh_3)_4$	–	K_2CO_3	Toluene + Ethanol + Water (3:1:1)	120	86.5% @18.0 h
1	$Pd(PPh_3)_4$	–	K_2CO_3	Toluene + Ethanol + Water (3:1:1)	100	31.0% @ 3.8 h
2	$Pd(PPh_3)_4$	–	K_2CO_3	THF + Water (3:1)	80	Not reacted
3	$Pd(PPh_3)_4$	–	K_2CO_3	DMF	150	62.9% @ 3.8 h
4	$Pd(dba)_2$	TPF	KOAc	Toluene + Ethanol + Water (3:1:1)	100	7.4% @ 1.0 h
5	$Pd(OAc)_2$	$P(o\text{-}tol)_3$	K_2CO_3	THF + Water (3:1)	80	Not reacted
6	$Pd_2(dba)_3$	BrettPhos	KOAc	THF + Water (3:1)	80	8.4% @ 3.5 h
7	$Pd(dba)_2$	TPF	K_2CO_3	Toluene + Ethanol + Water (3:1:1)	100	Not reacted
8	$Pd(OAc)_2$	TPF	KOAc	Toluene + Ethanol + Water (3:1:1)	100	Not reacted
9	$Pd_2(dba)_3$	BrettPhos	KOAc	Toluene + Ethanol + Water (3:1:1)	100	100% @ 3.7 h
10	$Pd(OAc)_2$	PPh_3	K_2CO_3	Toluene + Ethanol + Water (3:1:1)	100	35.7% @ 6.0 h
11	$Pd(OAc)_2$	PPh_3	K_2CO_3	DMF	150	Not reacted
12	$Pd(OAc)_2$	$PtBu_3$	K_2CO_3	DMF	150	23.8% @ 6.3 h
13	$Pd(PPh_3)_4$	–	Na_2CO_3	DMF	150	68.0% @ 6.0 h
14	$Pd(PPh_3)_4$	–	NaOH	DMF	150	Not reacted

* Size of search space = 2,722 = (4 catalysts × 19 ligands + 1 catalyst without ligand) × 9 bases × 4 solvents

Table S16. Autonomous synthetic conditions for M2.

Trial number	Catalyst	Ligand	Base	Solvent	Reaction temperature (℃)	Maximum conversion yield
Reference	$Pd_2(dba)_3$	JohnPhos	NaOtBu	Toluene	100	15.0% @ 3.5 h
1	$Pd(OAc)_2$	JohnPhos	K_2CO_3	DMSO	110	Not reacted
2	$Pd(OAc)_2$	BINAP	K_2CO_3	DMSO	110	Not reacted
3	$Pd(OAc)_2$	PPh_3	K_2CO_3	DMSO	110	Not reacted
4	$Pd_2(dba)_3$	JohnPhos	Na_2CO_3	DME	80	Not reacted
5	$Pd(dba)_2$	SPhos	K_3PO_4	o-Xylene	120	32.9% @ 9.1 h
6	$Pd(OAc)_2$	DavePhos	NaOtBu	Toluene	110	7.9% @ 6.1 h
7	$Pd(OAc)_2$	$PtBu_3$	K_2CO_3	DMSO	110	Not reacted
8	$Pd(OAc)_2$	PCy_3	K_2CO_3	DMSO	110	Not reacted
9	$Pd(OAc)_2$	APhos	K_2CO_3	DMSO	110	Not reacted
10	$Pd(OAc)_2$	$P(o\text{-}tol)_3$	K_2CO_3	DMSO	110	Not reacted
11	$Pd(OAc)_2$	PPh_3	NaOtBu	Toluene	110	Not reacted
12	$Pd(OAc)_2$	$PtBu_3$	NaOtBu	Toluene	110	11.8% @ 1.4 h
13	$Pd(OAc)_2$	APhos	NaOtBu	Toluene	110	12.8% @ 3.9 h
14	$Pd(OAc)_2$	$P(o\text{-}tol)_3$	NaOtBu	Toluene	110	Not reacted
15	$Pd(OAc)_2$	TPF	Cs_2CO_3	Toluene	110	Not reacted
16	$Pd(dba)_2$	TPF	NaOtBu	o-Xylene	120	Not reacted
17	$Pd(OAc)_2$	$PtBu_3$	K_2CO_3	Toluene	110	Not reacted
18	$Pd(dba)_2$	$PtBu_3$	K_3PO_4	o-Xylene	120	10.8% @ 6.3 h
19	$Pd(dba)_2$	DavePhos	K_3PO_4	o-Xylene	120	28.3% @ 6.5 h
20	$Pd(dba)_2$	PPh_3	K_3PO_4	o-Xylene	120	Not reacted
21	$Pd(dba)_2$	PCy_3	K_3PO_4	o-Xylene	120	Not reacted
22	$Pd(dba)_2$	SPhos	K_3PO_4	THF	70	12.1% @ 12.1 h
23	$Pd(dba)_2$	SPhos	K_3PO_4	DMF	150	40.8% @ 4.3 h
24	$Pd(dba)_2$	SPhos	K_3PO_4	MeCN	80	Not reacted
25	$Pd(dba)_2$	SPhos	K_3PO_4	1,4-dioxane	100	Not reacted
26	$Pd(dba)_2$	SPhos	K_3PO_4	Toluene	110	28.4% @ 15.0 h
27	$Pd(OAc)_2$	SPhos	K_2CO_3	DMF	150	11.6% @ 3.5 h
28	$Pd(OAc)_2$	SPhos	K_3PO_4	DMF	150	26.9% @ 3.9 h
29	$Pd_2(dba)_3$	SPhos	K_3PO_4	DMF	150	37.4% @ 3.5 h
30	$Pd(OAc)_2$	SPhos	K_3PO_4	o-Xylene	120	20.3% @ 3.9 h
31	$Pd_2(dba)_3$	SPhos	K_3PO_4	o-Xylene	120	33.2% @ 9.0 h
32	$Pd(OAc)_2$	SPhos	K_3PO_4	Toluene	110	20.2% @ 9.0 h
33	$Pd_2(dba)_3$	SPhos	K_3PO_4	Toluene	110	27.8% @ 12.3 h
34	$Pd(OAc)_2$	DavePhos	K_3PO_4	o-Xylene	120	21.6% @ 3.5 h
35	$Pd_2(dba)_3$	DavePhos	K_3PO_4	o-Xylene	120	32.3% @ 9.2 h
36	$Pd(dba)_2$	APhos	NaOtBu	Toluene	110	100.0% @ 1.0 h
37	$Pd_2(dba)_3$	APhos	NaOtBu	Toluene	110	100.0% @ 1.3 h
38	$Pd(OAc)_2$	SPhos	K_3PO_4	THF	70	12.5% @ 6.3 h
39	$Pd_2(dba)_3$	SPhos	–	THF	70	16.1% @ 9.1 h
40	$Pd(dba)_2$	PPh_3	NaOtBu	Toluene	110	11.2% @ 6.2 h
41	$Pd(dba)_2$	APhos	K_2CO_3	Toluene	110	5.7% @ 4.1 h
42	$Pd_2(dba)_3$	APhos	K_2CO_3	Toluene	110	Not reacted
43	$Pd(dba)_2$	$PtBu_3$	NaOtBu	Toluene	110	100.0% @ 1.4 h
44	$Pd_2(dba)_3$	$PtBu_3$	NaOtBu	Toluene	110	100.0% @ 1.4 h
45	$Pd(dba)_2$	PCy_3	NaOtBu	Toluene	110	16.3% @ 3.5 h
46	$Pd(dba)_2$	$P(o\text{-}tol)_3$	NaOtBu	Toluene	110	Not reacted
47	$Pd(dba)_2$	TPF	NaOtBu	Toluene	110	8.7% @ 6.4 h

48	$Pd(dba)_2$	XantPhos	NaOtBu	Toluene	110	100.0% @ 1.5 h
49	$Pd_2(dba)_3$	PPh_3	NaOtBu	Toluene	110	11.4% @ 3.9 h
50	$Pd_2(dba)_3$	PCy_3	NaOtBu	Toluene	110	36.3% @ 1.4 h
51	$Pd_2(dba)_3$	$P(o\text{-}tol)_3$	NaOtBu	Toluene	110	5.7% @ 1.4 h
52	$Pd_2(dba)_3$	$PtBu_3$	NaOtBu	THF	70	6.5% @ 3.9 h
53	$Pd_2(dba)_3$	XantPhos	NaOtBu	Toluene	110	100.0% @ 1.4 h
54	$Pd(OAc)_2$	XantPhos	NaOtBu	Toluene	110	100.0% @ 1.2 h
55	$Pd_2(dba)_3$	APhos	NaOtBu	THF	70	8.3% @ 3.6 h
56	$Pd(dba)_2$	XantPhos	NaOtBu	o-Xylene	120	100.0% @ 1.4 h
57	$Pd(dba)_2$	$PtBu_3$	NaOtBu	o-Xylene	120	100.0% @ 1.3 h
58	$Pd(OAc)_2$	PCy_3	NaOtBu	Toluene	110	7.7% @ 1.5 h
59	$Pd_2(dba)_3$	DavePhos	NaOtBu	Toluene	110	67.5% @ 3.9 h
60	$Pd_2(dba)_3$	JohnPhos	NaOtBu	Toluene	110	13.4% @ 3.8 h
61	$Pd(dba)_2$	SPhos	K_2CO_3	DMF	150	26.4% @ 3.8 h
62	$Pd(OAc)_2$	XantPhos	NaOtBu	o-Xylene	120	100.0% @ 1.2 h
63	$Pd(OAc)_2$	XantPhos	NaOtBu	DMSO	110	Not reacted
64	$Pd(OAc)_2$	$PtBu_3$	NaOtBu	o-Xylene	120	71.3% @ 1.4 h

* Size of search space = 4,104 = 3 catalysts × 19 ligands × 9 bases × 8 solvents

Table S17. Autonomous synthetic conditions for M3.

Trial number	Catalyst	Ligand	Base	Solvent	Reaction temperature (℃)	Maximum conversion yield
Modified reference	$Pd_2(dba)_3$	XPhos	NaOtBu	DME	90	50.9% @ 12.0 h
1	$Pd_2(dba)_3$	$PtBu_3$	K_2CO_3	Toluene	110	1.8% @ 6.3 h
2	$Pd_2(dba)_3$	SPhos	K_2CO_3	Toluene	110	2.5% @ 4.1 h
3	$Pd_2(dba)_3$	$P(o\text{-}tol)_3$	K_2CO_3	Toluene	110	4.9% @ 3.6 h
4	$Pd_2(dba)_3$	DavePhos	Cs_2CO_3	Toluene	110	65.5% @ 18.3 h
5	$Pd_2(dba)_3$	JohnPhos	K_3PO_4	Toluene	110	4.0% @ 3.5 h
6	$Pd_2(dba)_3$	PPh_3	NaOH	1,4-Dioxane	100	47.0% @ 3.9 h
7	$Pd_2(dba)_3$	PPh_3	K_2CO_3	Toluene	110	3.7% @ 3.9 h
8	$Pd_2(dba)_3$	JohnPhos	K_2CO_3	Toluene	110	4.9% @ 1.4 h
9	$Pd_2(dba)_3$	PPh_3	K_3PO_4	Toluene	110	15.0% @ 9.0 h
10	$Pd_2(dba)_3$	$P(o\text{-}tol)_3$	Na_2CO_3	Toluene	110	9.4% @ 1.0 h
11	$Pd_2(dba)_3$	$PtBu_3$	NaOH	1,4-Dioxane	100	34.2% @ 3.7 h
12	$Pd_2(dba)_3$	PCy_3	NaOH	1,4-Dioxane	100	0.4% @ 3.6 h
13	$Pd_2(dba)_3$	APhos	NaOH	1,4-Dioxane	100	56.6% @ 6.2 h
14	$Pd_2(dba)_3$	$P(o\text{-}tol)_3$	NaOH	1,4-Dioxane	100	6.2% @ 1.0 h
15	$Pd_2(dba)_3$	TPF	NaOH	1,4-Dioxane	100	Not reacted
16	$Pd_2(dba)_3$	PCy_3	K_2CO_3	Toluene	110	2.8% @ 3.9 h
17	$Pd_2(dba)_3$	DavePhos	Cs_2CO_3	DMF	150	52.4% @ 6.4 h
18	$Pd_2(dba)_3$	DavePhos	Cs_2CO_3	THF	70	9.5% @1.4 h
19	$Pd(OAc)_2$	DavePhos	Cs_2CO_3	Toluene	110	40.1% @ 9.3 h
20	$Pd(dba)_2$	DavePhos	Cs_2CO_3	Toluene	110	52.6% @ 6.5 h
21	$Pd_2(dba)_3$	DavePhos	Cs_2CO_3	MeCN	80	64.7% @ 24.1 h
22	$Pd_2(dba)_3$	XantPhos	NaOH	1,4-Dioxane	100	40.0% @ 6.0 h
23	$Pd(OAc)_2$	APhos	NaOH	1,4-Dioxane	100	24.0% @ 3.9 h
24	$Pd(dba)_2$	APhos	NaOH	1,4-Dioxane	100	33.7% @ 1.4 h
25	$Pd_2(dba)_3$	APhos	K_2CO_3	1,4-Dioxane	100	Not reacted
26	$Pd(OAc)_2$	PPh_3	Cs_2CO_3	Toluene	110	52.1% @ 15.3 h
27	$Pd(OAc)_2$	$PtBu_3$	Cs_2CO_3	Toluene	110	60.9% @ 12.7 h
28	$Pd(OAc)_2$	DavePhos	Cs_2CO_3	DMF	150	59.0% @ 6.0 h
29	$Pd(dba)_2$	DavePhos	Cs_2CO_3	DMF	150	56.8% @ 6.4 h
30	$Pd(OAc)_2$	PPh_3	NaOH	1,4-Dioxane	100	7.5% @ 4.0 h
31	$Pd(dba)_2$	PPh_3	NaOH	1,4-Dioxane	100	5.8% @ 3.6 h
32	$Pd(OAc)_2$	XantPhos	NaOH	1,4-Dioxane	100	35.5% @ 3.5 h
33	$Pd(dba)_2$	XantPhos	NaOH	1,4-Dioxane	100	40.2% @ 3.9 h
34	$Pd_2(dba)_3$	PPh_3	Cs_2CO_3	DMF	150	70.7% @ 12.4 h
35	$Pd_2(dba)_3$	$PtBu_3$	Cs_2CO_3	DMF	150	56.1% @ 6.0 h
36	$Pd_2(dba)_3$	PPh_3	Cs_2CO_3	Toluene	110	62.7% @ 15.0 h
37	$Pd(OAc)_2$	PCy_3	Cs_2CO_3	Toluene	110	46.5% @ 18.5 h
38	$Pd_2(dba)_3$	$PtBu_3$	Cs_2CO_3	Toluene	110	63.2% @ 9.3 h
39	$Pd(OAc)_2$	APhos	Cs_2CO_3	Toluene	110	8.9% @ 3.5 h
40	$Pd(OAc)_2$	$P(o\text{-}tol)_3$	Cs_2CO_3	Toluene	110	3.1% @ 4.0 h
41	$Pd(OAc)_2$	TPF	Cs_2CO_3	Toluene	110	28.3% @ 24.0 h
42	$Pd(OAc)_2$	XantPhos	Cs_2CO_3	Toluene	110	97.2% @ 24.0 h
43	$Pd(OAc)_2$	DPEPhos	Cs_2CO_3	Toluene	110	28.2% @24.0 h
44	$Pd_2(dba)_3$	PCy_3	Cs_2CO_3	Toluene	110	39.2% @ 24.0 h
45	$Pd_2(dba)_3$	TPF	Cs_2CO_3	Toluene	110	38.2% @ 24.0 h
46	$Pd_2(dba)_3$	XantPhos	Cs_2CO_3	Toluene	110	43.5% @ 9.0 h

47	$Pd_2(dba)_3$	DPEPhos	Cs_2CO_3	Toluene	110	57.0% @ 24.0 h

* Size of search space = 4,104 = 3 catalysts × 19 ligands × 9 bases × 8 solvents

Table S18. Additional synthetic conditions for M3 using NaOtBu.

Trial number	Catalyst	Ligand	Base	Solvent	Reaction temperature (℃)	Maximum conversion yield
1	$Pd(OAc)_2$	$PtBu_3$	NaOtBu	Toluene	110	1.9% @ 3.8 h
2	$Pd(OAc)_2$	XPhos	NaOtBu	Toluene	110	4.2% @ 3.8 h
3	$Pd(OAc)_2$	XantPhos	NaOtBu	Toluene	110	69.0% @ 1.0 h
4	$Pd_2(dba)_3$	$PtBu_3$	NaOtBu	Toluene	110	1.9% @ 3.9 h
5	$Pd_2(dba)_3$	XPhos	NaOtBu	Toluene	110	64.7% @ 3.5 h
6	$Pd_2(dba)_3$	XantPhos	NaOtBu	Toluene	110	71.8% @ 1.0 h

Movie S1.

Working procedure of the Synbot.

Data S1. (separate file)

Raw data for the autonomous synthesis of M1–M3
